# Improving morphology and optoelectronic properties of ultra-wide bandgap perovskite via Cs tuning for clear solar cell and UV detection applications

**DOI:** 10.1038/s41598-023-29409-y

**Published:** 2023-02-20

**Authors:** Myo Zin Tun, Pimsuda Pansa-Ngat, Pipat Ruankham, Ko Ko Shin Thant, Sirawit Kamnoedmanee, Chaowaphat Seriwattanachai, Worawut Rueangsawang, Ratchadaporn Supruangnet, Hideki Nakajima, Pongsakorn Kanjanaboos

**Affiliations:** 1 School of Materials Science and Innovation, Faculty of Science, Mahidol University, Nakhon Pathom, 73170 Thailand; 2 Department of Physics and Materials Science, Faculty of Science, Chiang Mai University, Chiang Mai, 50200 Thailand; 3 Research Center in Physics and Astronomy, Faculty of Science, Chiang Mai University, Chiang Mai, 50200 Thailand; 4 Synchrotron Light Research Institute (Public Organization), Nakhon Ratchasima, 30000 Thailand; 5 Center of Excellence for Innovation in Chemistry (PERCHCIC), Ministry of Higher Education, Science, Research and Innovation, Bangkok, 10400 Thailand

**Keywords:** Solar cells, Devices for energy harvesting, Electronic devices

## Abstract

With growing population, vertical spaces from skyscrapers are vast. Semi-transparent solar cells enable an effective pathway for vertical energy harvesting. With composition tunability, perovskite materials can be designed with different transparencies and colors. In this work, an ultra-high bandgap layered triple cation perovskite system was developed for the first time to meet the demand of clear optoelectronic applications; low dimensional triple cation perovskite thin films were fabricated using perovskite with the formula (PEA)_2_(Cs_x_MA_0.61-x_FA_0.39_)_39_(Pb)_40_(Cl_0.88-0.32x_Br_0.12+0.32x_)_121_, 0 ≤ *x* ≤ 0.02 with DMSO as the appropriate solvent. The absorption edge of the material is around 410–430 nm, achieving great transparency to visible light. The structural, optical, and photovoltaic performances of the clear perovskite materials are explored with the variation of Cs contents via CsBr. The relation between thickness, transparency, and optoelectronic properties of the clear perovskite materials along with other physical properties were investigated. The highest photovoltaic conversion efficiency (PCE) of clear perovskite solar cells with 1.5% Cs was achieved to be 0.69% under xenon lamp irradiation at 100 mW/cm^2^ (1.5 mW/cm^2^ of UVA within 100 mW/cm^2^) and 5.24% under 365 nm UV irradiation at 2.4 mW/cm^2^. Photoresponsivity, external quantum efficiency (EQE), and detectivity were also determined for photodetector applications.

## Introduction

Perovskite solar cells, which have superior properties as high absorption coefficient and high-power conversion efficiency along with low-cost fabrication, have come under the spotlight^1–7^. Semi-transparent/transparent perovskite solar cells have the potential to supplement the commercial building integrated photovoltaics^8^. Emergence of 3D perovskite has attracted much attention due to remarkable performance; however, 3D perovskite structure (ABX_3_) comes short with structural, thermal, and moisture instability^9–11^. For stability improvement, dimensional tuning of 3D structure to quasi-2D by inserting a large organic cation such as PEA^+^ or BA^+^ was demonstrated^12–14^. Unlike two dimensional perovskites, quasi-2D structures with highly controlled stacked layers and large *n* values have not only promising stability but also acceptable optoelectronic properties^15–17^. Regardless of having high stability to moisture, poor interlayer charge transport, high exciton binding energy, and large energy bandgap were observed in quasi-2D structures^18^. Another solution for both stability and efficiency enhancement is the triple cation system with Cs, MA, and FA in specific ratios. The MA/FA mixture without Cs can enhance the efficiency, but the occurrence of photo-inactive yellow δ-phases is frequently observed^18–20^. Comparing to MA/FA, ionic radius of Cs is smaller and the mismatch between ionic radii of Cs and FA leads to lattice distortion resulting in rapid changes of lattice parameters and non-linear shifting^21^. With Cs incorporation, strong interaction within Cs, FA, MA, and halides occur, initiating the reduction of unit cell volume where the presence of stable α phase can be found at lower temperature^22^. Due to lattice distortion, Cs incorporation becomes thermodynamically stable, having better thermal and moisture resistivity and less probability of converting into the δ-phase; additionally, lower trap state density leads to longer minority lifetime, less recombination, and therefore better efficiency^10,23,24^. However, upon Cs incorporation, to achieve a stable phase, the relation between phase stabilization, entropy contributions, and Gibbs free energy is needed to be considered. As theoretically and practically stated, in the stabilization of perovskite phases, the entropy contribution to the Gibbs free energy driven by isotropic rotations of the FA is crucial^25^. The reduction of Gibbs free energy occurs with small Cs content, enhancing the stability of α-phase; however, excessive cation mixing could result in higher Gibbs free energy due to the movement of FA cations, which promotes phase segregations and δ-phase formation^25–27^. Structural stability of perovskite materials is usually considered by tolerance factor where 0.81 < t < 1.11 is considered to be stable. As stated, Cs, FA, MA mixture promotes an increase in tolerance factor, theoretically more than 20% of Cs incorporation has the most stable phase, although inhomogeneous Cs_4_PbX_6_ phases emerge in conditions where Cs^+^ content is more than 37.5%^28^. Outdoor perovskite solar cells require efficiencies of more than 20% to compete with those of silicon counterparts. However, as a window replacement, transparency is more crucial for clear perovskite photovoltaics. With mainly UV absorption, the efficiency of less than a percentage is expected. Therefore, perovskite with bandgap beyond 2.9 eV can be an innovative platform for transparent solar cells, where absorption, transmission, and photoconversion can be realized at the same time^29^. As a solar cell is one kind of photodiode, large bandgap perovskite can also be analogously used as a UV sensor^30^. Previously, wider bandgap perovskite in a single crystal form exhibited high responsivity under UV irradiation along with detectivity close to 1.2 × 10^10^ cmHz^½^W^−1^^31,32^. The clear solar cells fabricated by the thin film technology which combines respectable PCE, longer lifetime, with higher transparency are rarely reported^33^. The transparent films can be achieved by anion substitution, bandgap tuning, and thickness alteration; to date; respectable PCE from 0.1 to 0.5% with transparency between 50 to 70% can be achieved^29,34^. Smooth perovskite morphology and good surface coverage are imperative for repeatability and scalability; however, there is no figurative evidence of such in previous studies^29,30,35–37^. Morphology and crystallization of perovskite materials are highly dependent on processing parameters such as coating speed and duration, drying conditions such as annealing temperature and time, and the use of solvents such as solvent and anti-solvent types and qualities^27^. In general, using the appropriate solvent for perovskite precursor solution is important as the morphology is largely governed by solvents. The choice of the solvent is considered with its solubility, usually decided by Gutmann’s donor number (D_N_)^38^. As stated before, solvents with high D_N_ values have high solubility to dissolve perovskite precursor and have stable bonding with Pb^2+^; comparing between the commonly used aprotic solvents; DMSO has higher a D_N_ value compared to that of DMF^39,40^. DMF-based films exhibited rough surface along with cracks and pinholes, meanwhile DMSO-based films shows good coverage along with smaller grains, while DMF/DMSO mixture reported notable smooth morphology^40,41^. PbCl_2_ is a major component in all most reported ultra-wide bandgap perovskite solar cell; however, due to its low solubility compared to PbI_2_ and PbBr_2_^42,43^, PbCl_2_ most likely leads to rapid crystallization and therefore high roughness. In agreement with previous studies, the surfaces of ultra-wide bandgap are inhomogeneous, rough, exhibiting scattered flake-like structures with the use of DMF:DMSO (3:1) or DMSO as the solvents^44^. However, smooth and uniform morphology were demonstrated with a vacuum-assisted solution deposition process, followed by methylamine gas post-treatment^29,44^. Alternatively, small incorporation of Cs can enhance the morphology^45^. More than 10% of Cs incorporation in triple cation perovskites in DMF:DMSO (4:1) led to less coverage and pinholes, which resulted in poor photovoltaics performance, yet pinhole-free, uniform films were achieved when Cs incorporation is less than 5%^46^. Another report observes crystal size enhancement and compact surface morphology with Cs inclusion up to 2%^45^.

To the best of our knowledge, there are only a few publications to date, mainly describing the use of MAPbCl_3_-based thin films for transparent solar cells. In this work, we, for the first time, combine the protective capacity of Quasi-2D, the performance of triple cation formulae, and the applicability of wide bandgap materials, introducing﻿ (PEA)_2_(Cs_x_MA_0.61-x_FA_0.39_)_39_(Pb)_40_(Cl_0.88-0.32x_Br_0.12+0.32x_)_121_, 0 ≤ *x* ≤ 0.02 as a material for transparent solar cell. As PbCl_2_ has a higher solubility in DMSO compared to DMF, DMSO was used as the only solvent. With 1.5% Cs, a bandgap of 2.9 eV and champion PCEs of 0.69% and 5.24% under one sun and 365 nm UV irradiation, respectively, along with the transparency of 61% are demonstrated. More importantly, smooth perovskite morphology and good device repeatability can be achieved with the 1.5% Cs addition. The device was also tested for its UV sensing aptitude. With decent morphology and coverage, the perovskite formula opens doors for large-scale solar windows in the future.

## Methodology

Lead(II) chloride (PbCl_2_; ≥ 98%), lead(II) bromide (PbBr_2_; ≥ 98%), formamidine hydrochloride (FACl; ≥ 97%, anhydrous), methylamine hydrochloride (MACl; ≥ 98%), cesium bromide (CsBr; 99.9%), phenethylammonium bromide (PEABr; 99.95%), anhydrous dimethyl sulfoxide (DMSO; 99.9% v/v), ethyl acetate (EA; ≥ 99.5%), anhydrous ethanol (ETOH; 99.5% v/v), titanium dioxide (TiO_2_; 99.999%), and hydrochloric acid (HCl; 37% v/v) were purchased from Sigma-Aldrich.

0.3 M of TiO_2_ was prepared by adding 780 µL of titanium(IV) isopropoxide slowly into 8 mL of anhydrous ethanol while stirring. 60 µL of 37% hydrochloric acid was added into the solution and stirred for overnight. The precursor solution was deposited on cleaned FTO, which was treated with UV-ozone for 30 min, followed by spinning at 2000 rpm with ramp rate of 1000 rpm/s for 30 s. The deposited films were annealed step by step at 125 °C, 255 °C, and 375 °C. Annealing time for each step is 15 min. Then, the temperature was increased to 520 °C for the 1 hr annealing process before cooling down. The deposition of *c*-TiO_2_ film was done inside the glovebox and annealing process was done outside of the glovebox. With 2 M concentration, 6.6 mL of TiCl_4_ is dropped into 30 mL of frozen DI water; later, the TiCl_4_ solution was diluted to be 0.12 M. The diluted solution was slowly poured to the substrates and baked at 70 °C for 30 min. The substrates were rinsed 3 times with DI water and N_2_ gas was used for drying. They were then dried at 70 °C for 30 min. After that, the temperature was increased to 520 °C for the 2 hr annealing process. The substrates were cooled down to room temperature. TiCl_4_ treatment was done outside of the glovebox.

The transparent low dimensional triple cation perovskites were fabricated using formula of (PEA)_2_(Cs_x_MA_0.61-x_FA_0.39_)_39_(Pb)_40_cl_0.88-0.32x_Br_0.12+0.32x_)_121_ where *x* = 0, 0.005, 0.01, 0.015, and 0.02 for 0%, 0.5%, 1%, 1.5%, and 2% Cs, respectively, which are quasi-2D (*n* = 40) and *x* is according to the stoichiometric ratio from input precursors. CsBr (21.28 mg) was weighed and mixed with DMSO (1 mL) for a stock precursor (0.1 M). For example (to make x = 0.015 or 1.5% Cs at 0.8 M), the weights of each powder for 1 mL solution are as follows: PbCl_2_ (189.11 mg), PbBr_2_ (44.04 mg), MACl (31.33 mg), FACl (24.47 mg), and PEABr (8.08 mg) are weighed together in a vial. Then, CsBr (117 µL, 0.1 M) from the stock solution and DMSO (883 µL) were added and mixed with the powder. Then, the solution was stirred at 70 °C for 30 min. Too much stirring time period and heat typically lead to undesirable results. Then, after 30 min of stirring, color of the precursor solution turned into clear, transparent color, without any powder residues inside. Then the solution was filtered with a PTFE syringe filter. For other conditions: 0% Cs, 0.5% Cs, 1.0% Cs, and 2.0% Cs, the mass of MACl, the volume of CsBr, and the volume of DMSO are different based on the stoichiometric ratios, while the mass of PbCl_2_, PbBr_2_, FACl, and PEABr were kept the same as the case of 1.5% Cs. To prepare the clear, transparent perovskite thin film, 100 µL of precursor solution was dropped onto an etched *c*-TiO_2_ substrate, which was treated with UV-ozone for 30 min prior and spun at 750 rpm (ramp rate = 375 rpm/s) for 11 s, followed by a second spinning step of 3000 rpm for 30 s (ramp rate = 1500 rpm/s). During the spinning process, 250 µL of ethyl acetate (EA) was quickly dropped after 27 s from the start of the spin process. The deposited perovskite film was then annealed at 100 °C for 15 min. The experiment was carried out inside N_2_-filled glovebox.

Spiro-OMeTAD solution was prepared by dissolving 80 mg spiro-OMeTAD in 1 mL chlorobenzene, then 28.5 µL of 4-tertbutyl pyridine was added; the solution was then stirred at room temperature for 30 min. 1.81 M of Li-TFSI stock solution was prepared by adding 520 mg of Li-TFSI salt to 1 mL of acetonitrile. Lastly, 17.5 µL of the Li-TFSI solution was added into the hole transport layer mixture. The solution was stirred inside N_2_-filled glovebox at room temperature for 12 h. The greenish transparent color was observed. 50 µL of solution was dropped onto the perovskite layer, rested for 30 s, and then spun at 2000 rpm (ramp rate = 1000 rpm/s) for 35 s. Then, samples were rapidly exposed to ambient air for 20 s and returned to N_2_-filled glovebox for aging overnight. For thermal evaporation of gold (Au) and silver (Ag), 15 nm of Au (0.2 A°/s) and 65 nm of Ag (1.3 A°/s) were deposited through an evaporation mask to get individual cell areas of 0.049 cm^2^ at a pressure of ≈10^−6^ mbar in a thermal evaporator. The temperature of thermal evaporator chamber was carefully controlled to be at 40 °C or below during the evaporation.

## Characterization methods

Fabricated transparent clear perovskite films were studied by different characterization methods. The crystal structure and the stability tracking of perovskite films were studied by X-ray diffractometer (Cu anode material, detector scan mode using a step size of 0.02°, 198.7318 s per step, and 2θ from 5° to 50°). Ultraviolet photoelectron spectroscopy was measured at beamline 3.2 Ua/b at Synchrotron Light Research Institute (public organization), Thailand. The absorption spectra were obtained by using Cary 60 UV–Vis spectrophotometer (300–﻿600 nm, slow scan, absorbance mode, and transmittance mode). The photoluminescence (PL) spectra were measured by Horiba FluoroMax4 + spectrofluorometer (integration time of 1.0 s, excitation of 330 nm, excitation slit of 8 nm, emission wavelength measurement between 365 and 620 nm, and emission slit of 8 nm). Surface morphologies and cross-sections were observed by field scanning electron microscopy (FESEM; JSM-7610Fplus JEOL, 10 kV, and secondary electron mode). The topological images were performed by Park NX10 atomic force microscopy (AFM) using a contact probe (Forta, k = 3.4 N/m, and resonance frequency = 60 kHz). The measurement setup was done with a contact force setpoint of 19.45 nN and a scan speed of 1.5 μm/s. All the samples were measured in ambient air (~ 60%RH) at room temperature (~ 25 °C). One-sun irradiation (100 mW/cm^2^) was provided by a xenon lamp housed inside Oriel 66002. The light intensity was calibrated by Si-diode (Hamamatsu S1133). UV-irradiation measurements were performed by LLS-365 UV lamp (2.4 mW/cm^2^). EQE, responsivity, and specific detectivity were measured using Enlitech QE-R quantum efficiency analyzer (DC mode with 1 mm^2^ beam diameter). For photo-response measurements, the PPDs were exposed to one sun irradiation; an optical chopper spinning at 100 Hz was used to block the light path while the photovoltages of samples over 100 kΩ were measured with the Tektronix TBS1072b-EDU oscilloscope. Solar cell parameters including J-V curves were measured by a Keithley 2400 source meter unit. UV power intensities for different light sources were measured by Digicon (UV-370SD-UVA, UVC light meter). LLS-365 UV lamp and xenon lamp spectra were recorded by Ocean optics (USB 4000).

## Results and discussion

**Figure 1:**
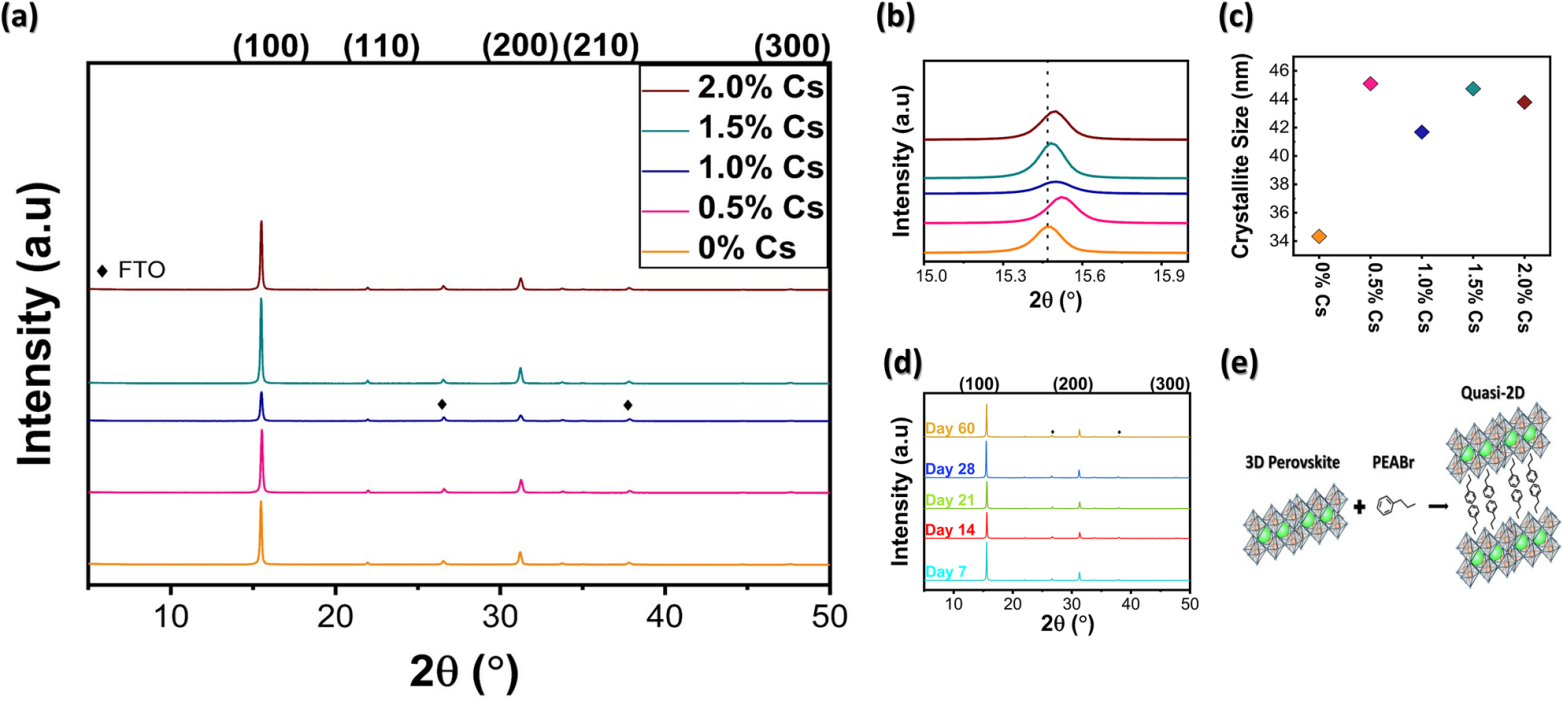
(**a**) XRD patterns of (PEA)_2_(Cs_x_MA_0.61-x_FA_0.39_)_39_(Pb)_40_(Cl_0.88-0.32x_Br_0.12+0.32x_)_121_ with different Cs contents. (110) and (210) planes belong to small amount of FAPbCl_3_. (**b**) XRD spectra of 0% Cs, 0.5% Cs, 1.0% Cs, 1.5% Cs, and 2.0% Cs at the 2θ from 15° to 16°. (**c**) Crystallite sizes of 0% Cs, 0.5% Cs, 1.0%, 1.5% Cs, and 2.0% Cs. (**d**) XRD spectra of 1.5% Cs showing stability of 60 days in moisture environment. (**e**) Diagram of quasi-2D, low-dimensional perovskite structure.

The diffraction peaks were well found at the positions of 15.46°, 31.32°, and 47.48°, corresponding to cubic or quasi-cubic structure with phase indexes of (100), (200), and (300), respectively, as shown in Fig. 1a, consistent with the other literature reports^3,28^. Although our perovskite materials’ dimension was altered to be quasi-2D (n = 40) in the triple cation system, the XRD patterns still confirm the cubic structure and feature planes of 3D counter parts in agreement with other reports^29,44^. In addition, the XRD intensity tends to increase with higher Cs amount. However, the non-linear shifting has occurred for (100) peaks from 15.46° to 15.56° as shown in Fig. 1b, due to lattice distortion from lattice mismatch between Cs, FA, and MA along with lattice reduction as the peaks tend to shift to higher angles^21^. This shift signals strong interaction within Cs, FA, MA, and halides upon Cs doping. Additionally, an appearance of inhomogeneous Cs_4_PbX_6_ phase is not found upon CsBr doping, proving that α-phase is formed without impurity^28,40^. Through Scherrer equation, the crystallite size calculation yields 34 nm, 45 nm, 41 nm, 44 nm, and 43 nm as indicated in Fig. 1c. Overall, increase in Cs content leads bigger crystallite size.

Since an organic large cation, PEA^+^, is inserted as the spacer cation to confine the dimension to be a quasi-2D structure with n = 40 as shown in Fig. 1e, the stability is supposedly enhanced^47,48^. To identify stability of perovskite films with different amounts of Cs, XRD measurements were performed for all samples stored at 60%RH at different time durations. Figure 1d gives the durability information for 1.5% Cs; the lack of a new peak even after 60 days proves elevated structural stability, suitable for future commercialization. Similar robustness is observed for the other Cs amounts, as shown in Fig. S1. Higher stability is attributed to strong interaction presented from Cs incorporation into MA and FA. However, materials degradation still occurred under a high temperature condition of 80–100 °C in Fig. S2.

**Figure 2:**
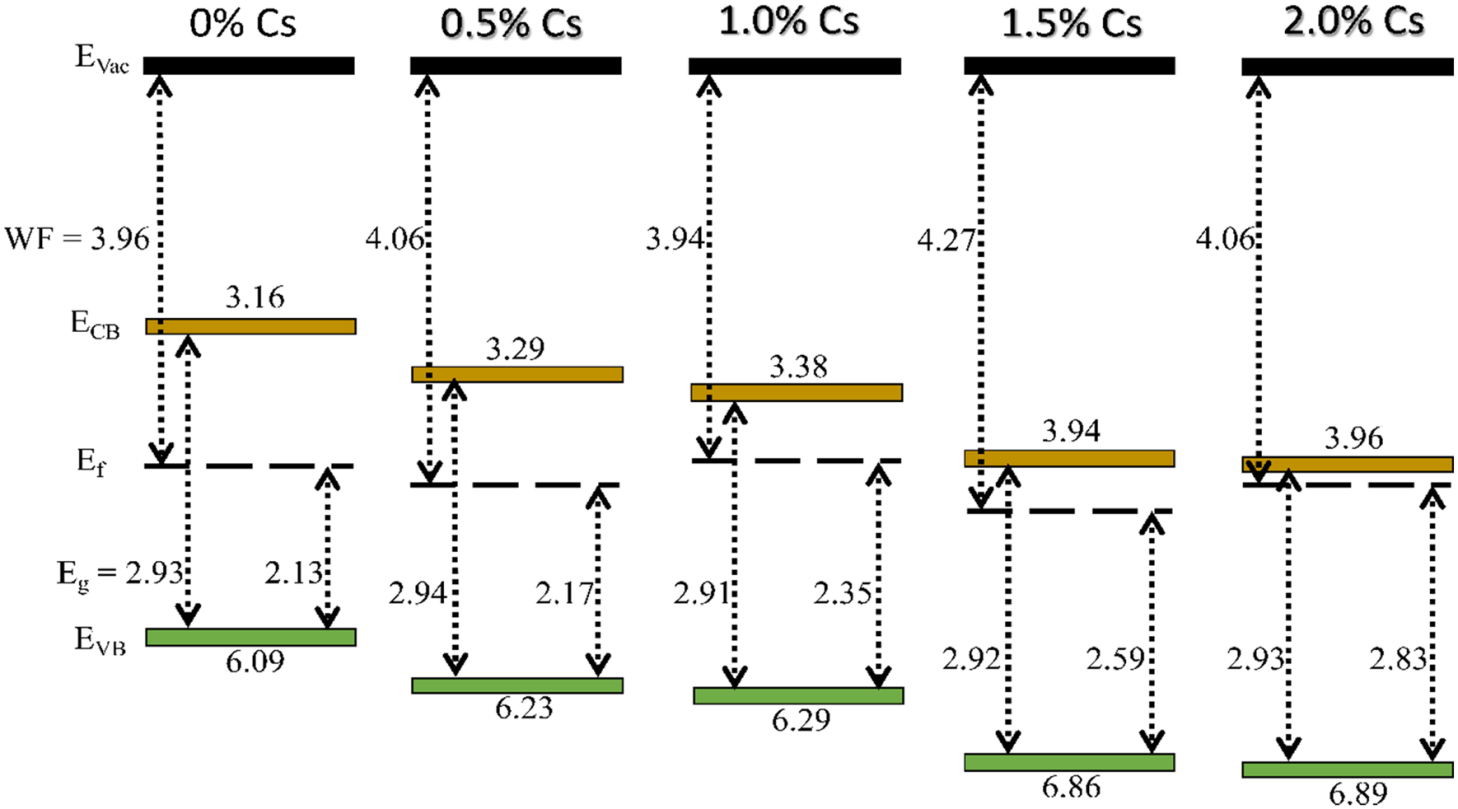
Band diagram of 0.8 M (PEA)_2_(Cs_x_MA_0.61-x_FA_0.39_)_39_(Pb)_40_(Cl_0.88-0.32x_Br_0.12+0.32x_)_121_ with 0% Cs, 0.5% Cs, 1.0% Cs, 1.5% Cs, and 2.0% Cs.

To clearly understand the effect of Cs doping on band structure of perovskite, UPS measurement was conducted. According to the UPS results, work functions of 0.5%, 1%, and 2% Cs are not much different from without doping one except 1.5% Cs. At 1.5% Cs as compared to 0% Cs, the valence band maximum (VBM) reduces from −6.09 eV to −6.86 eV along with increased work functions from 3.96 eV of 0% Cs to 4.27 eV and slight shifting of the bandgap. In previous reports, Cs incorporation shows the dominant effect to the band position of the perovskite material; this downward shift in VBM of Cs-doped perovskite is due to the loss of electron^49^. At high level of Cs doping, the VBM values become more negative, resulting in larger quasi-Fermi energy difference between VBM of perovskite and conduction band minimum (CBM) of electron transport material and therefore higher V_oc_^50^. This downward shift in VBM also contributes more driving force for hole transportation^51^. However, it should be noted that too much downshifting can result in mismatch in conduction band minimum alignment for electron transport, pointing the suitability of moderate Cs content. The band alignments are stacked, assuming the vacuum level as zero in Fig. 2; more details are given in Table S1. When perovskite layers make electrical contacts with transport layers, Fermi levels become aligned. As a result, CBM of 1.5% Cs matches well with CBM of TiO_2_ than that of other Cs conditions, indicating more charge carrier extraction especially for electron^52^. Band diagram of 1.5% Cs, aligned by having vacuum level equals to zero, are shown in Fig. S3.

**Figure 3:**
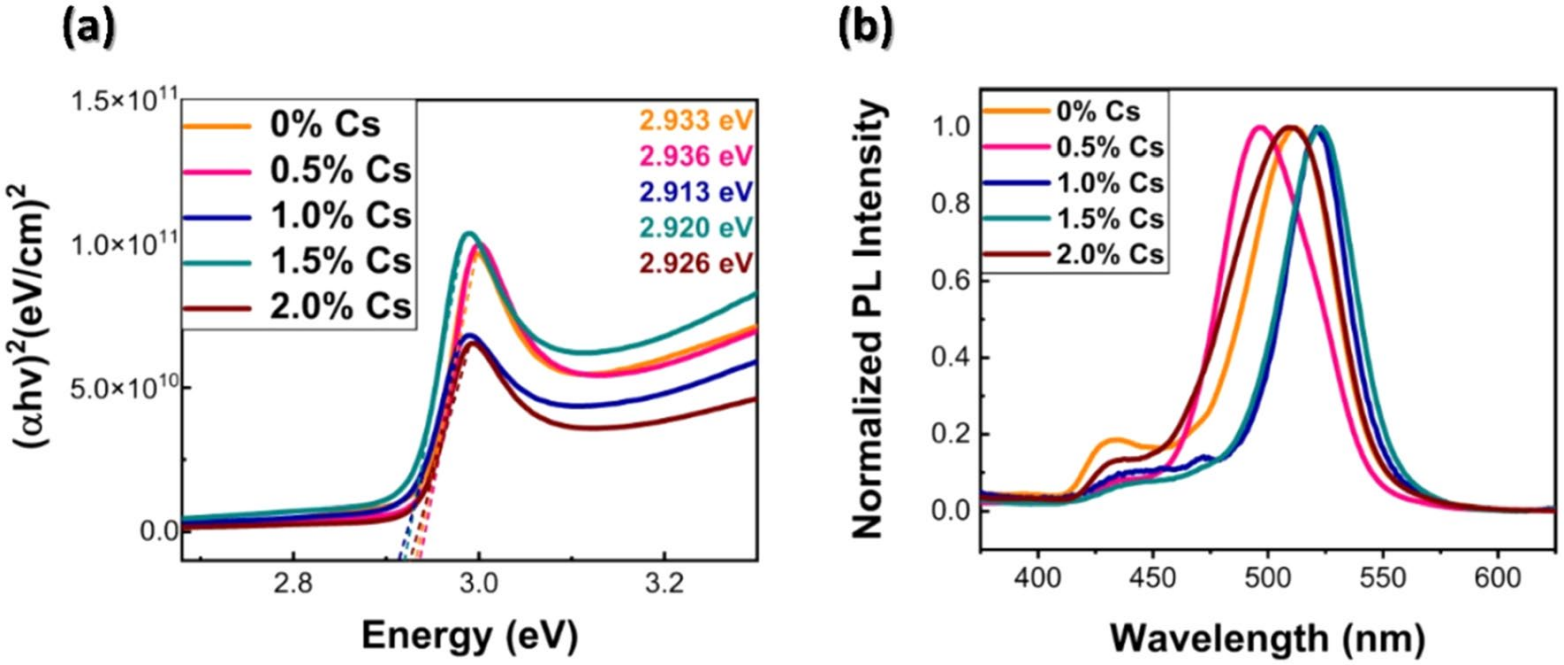
(**a**) Tauc Plot for optical bandgap (E_g_) analysis, and (**b**) PL Spectra of 0.8 M (PEA)_2_(Cs_x_MA_0.61-x_FA_0.39_)_39_(Pb)_40_(Cl_0.88-0.32x_Br_0.12+0.32x_)_121_ with 0% Cs, 0.5% Cs, 1.0% Cs, 1.5% Cs, and 2.0% Cs.

Then we studied the optical properties of (PEA)_2_(Cs_x_MA_0.61-x_FA_0.39_)_39_(Pb)_40_(Cl_0.88-0.32x_Br_0.12+0.32x_)_121_ films by using the UV–Vis spectroscopy and photoluminescence spectroscopy. Adding Cs into the structure has an influence on optical and structural properties^53^. Small amount of Cs doping does not have significant influence on the change of optical bandgap ~ 0.01–0.02 eV in Fig. 3a and Fig. S4. Our finding also agrees with previous reports; with small amount of Cs doping, no significant changes occur^45,54^. The obtained bandgaps are suitable for application for UV absorbing transparent photovoltaics, which can absorb the UV light by allowing the visible light source to pass through its layer^55^. Despite having the absorption cutoff at 410–430 nm, the photoluminescence spectra show the large Stokes shift; the PL peaks occur near 525 nm wavelength, > 100 nm shift from absorption edge. The Stokes shift is defined as the difference between the absorption edge and the emission peak maxima^56^, resulting from the relaxation of the lattice around the excitation state^4^, As stated, the presence of Cs_4_PbX_6_ phase in CsPbBr_3_ may cause large Stokes shift^57^. In our case, large Stokes shift appears in all conditions due to dimensional confinement, and some literatures have reported its dependency on the low dimensional phase^18,58–61^. The PL data of film prepared without dimensional confinement (PEABr) is shown in the supplementary information, Fig. S5 indicates noticeably less Stokes shift. The non-linear PL peak shifting occurs upon increasing Cs content, as in same trend with XRD. Also, in PL, another emission peak has occurred around 430 nm, exhibiting self-trapping which is usually observed in Pb-Br hybrid perovskites and induces the lattice distortion^62^. However, those extra peaks disappear as shown in Fig. 3b as Cs incorporation reaches to 1.5%, suggesting that the uniform distribution of Cs leads to phase formation without impurity.

**Figure 4:**
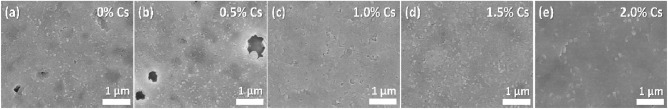
FESEM morphology images of 0.8 M (PEA)_2_(Cs_x_MA_0.61-x_FA_0.39_)_39_(Pb)_40_(Cl_0.88-0.32x_Br_0.12+0.32x_)_121_ with (**a**) 0% Cs, (**b**) 0.5% Cs, (**c**) 1.0% Cs, (**d**) 1.5% Cs, and (**e**) 2.0% Cs.

**Figure 5:**
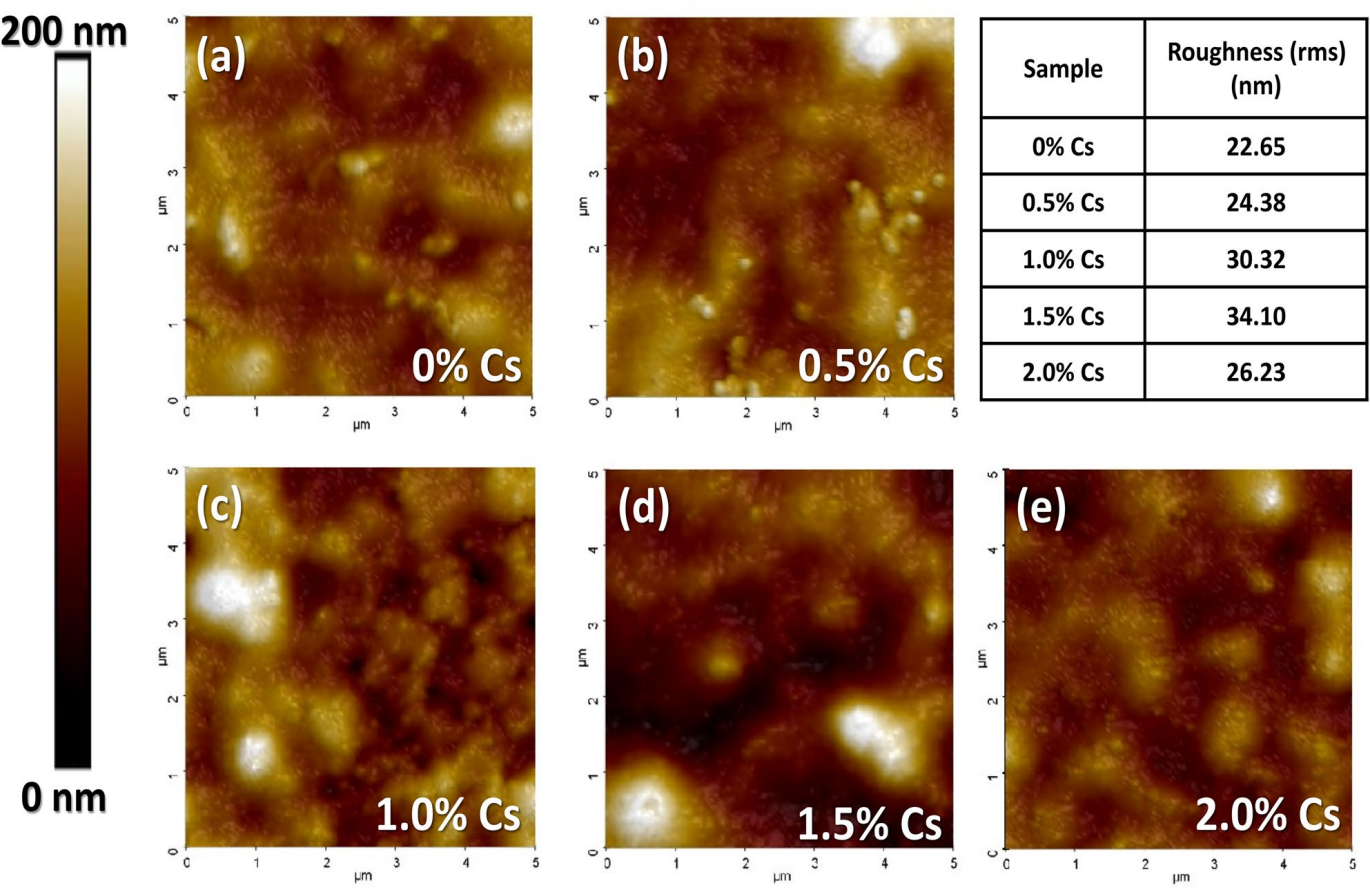
AFM morphology images of 0.8 M (PEA)_2_(Cs_x_MA_0.61-x_FA_0.39_)_39_(Pb)_40 _(Cl_0.88-0.32x_Br_0.12+0.32x_)_121_ with (**a**) 0% Cs, (**b**) 0.5% Cs, (**c**) 1.0% Cs, (d) 1.5% Cs, and (**e**) 2.0% Cs. The AFM scan size is 5 μm × 5 μm for all conditions.

Since the good coverage of perovskite morphology is required for better performances, the Cs doped films were treated with the anti-solvent (EA) during the spinning process and the morphology of the Cs doped films were analyzed by FESEM and pinpoint mechanical AFM techniques. The anti-solvent treatment helps boost homogeneity and fast nucleation at the surface^63^. In our case, EA removes the excess DMSO solvent which results in uniform morphology with good coverage. However, the morphology of the films differs from different Cs doping amount. As we can see from FESEM results in Fig. 4, the film coverage increases with an increase in Cs amount. We can see some defects and pinholes for the films with 0% Cs, 0.5% Cs and 1.0% Cs; however, 1.5–2.0% Cs leads to uniform morphology. As a result, Cs incorporation affects perovskite film formation and therefore repeatability of solar devices. From AFM images (scan area: 5 μm x 5 μm),  the incorporation of Cs does not have much impact on the roughness as shown in Fig. 5. Without Cs, the roughness is ~ 23 nm. With 1.5% Cs, it slightly increases to 34.1 nm. According to our findings, and in agreement with others, inhomogeneous distribution of Cs may cause the defects in morphology whether doping level is low or high^27,64^. However, the roughness in this work is comparatively less compared to previous work^71^. For comparison, morphologies of perovskite films prepared in DMF: DMSO (4:1) annealed at 100 °C for 15 min and morphologies of films prepared in DMSO solvent, annealed at 125 °C for 15 min are shown in Fig. S6, demonstrating the morphological dependency of solvent solubility. Along with FESEM images, the AFM surface modulus mapping of the films are described in Fig. S7. Cs doping does not really affect modulus distribution. The solubilities of different solvent types are also given in Table S2.

After careful analyzing of Cs doping variation, 1.5% Cs condition is our focus for further solar performances. The transparency of the solar cells depends on the thickness and the film coverage of the active layer. However, the balance between transparency and photovoltaics performance of the solar cells is needed^65,66^. The active perovskite layer is prepared as an ultra-thin layer to control the visible light absorption^67^. The average visible transmittance (AVT) is the common way of deciding whether the material is acceptable to be used as the transparent solar films^68^. We used the following equation to calculate the AVT from transmittance spectra, where λ is the wavelength, P(λ) is photopic response for human eyes at λ, S(λ) is solar flux (AM1.5G) at λ, and T(λ) is the transmittance at λ^66^.

1 $$AVT= \frac{\int T\left(\lambda \right)\,P\left(\lambda \right) \,S\left(\lambda \right)\,d(\lambda )}{\int P\left(\lambda \right)\, S\left(\lambda \right)\, d(\lambda )}.$$

**Figure 6:**
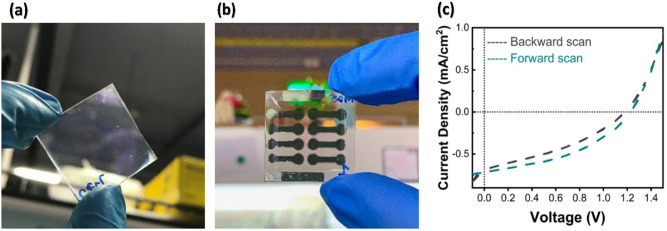
(**a**) Transparent film image, (**b**) Full device structure (FTO/*c*-TiO_2_/1.5% Cs/Spiro-OMeTAD/Au), and (**c**) Current–voltage curve of 0.8 M.

Our calculated AVT underpins the ideas of the inversed relationship between AVT and thicknesses (along with efficiency). This trend is also consistent with transmittance trend, given in the Fig. S8, and cross-section images of 1.5% Cs with concentration variation (0.3 M, 0.6 M, 0.8 M, 1.0 M, and 1.2 M) are given in Fig. S9. AVT and PV performances with thickness variation at 1.5% Cs are shown in Table 1. Clearly, thicker films are not suitable for places where visionary is important, yet they could go further in photodetector applications^29^. Despite having the AVT of 61%, the champion device of 0.8 M condition has enhanced efficiency of 0.69%, a V_oc_ of 1.1 V, a J_sc_ of 0.99 mA/cm^2^, and FF of 0.63. We believe that the lack of pinholes, defects, and good coverage that 1.5% Cs provides bring about less charge recombination, higher V_oc_, and higher efficiency^69,70^. Higher R_sh_ values from photovoltaics measurements are also obtained. For comparison, AVTs, thicknesses, and photovoltaics parameters for previously reported clear solar cells are shown in Table 2. The transparent film image, full device image, and J-V curves of our clear solar cells are shown in Fig. 6a–c, respectively.

**Table 1:** AVT and average PV performances for different concentrations and thicknesses at 1.5% Cs under one sun.

Concentration	V_oc_ (V)	J_sc_ (mA/cm^2^)	FF	PCE (%)	AVT (%)	Thickness (nm)	R_sh_ (ohm.cm^2^)
1.2 M	0.65	1.03	0.69	0.46	56.14	470	128,496
1.0 M	1.10	0.66	0.59	0.36	59.60	380	56,207
0.8 M	0.91	0.83	0.61	0.46	61.62	305	21,476
0.6 M	0.47	0.56	0.56	0.15	66.23	285	19,882
0.3 M	0.63	0.53	0.51	0.17	76.00	175	7410

**Table 2:** Comparison of PV performances for different reported clear solar cells under one sun.

Material	V_oc_ (V)	J_sc_ (mA/cm^2^)	FF	PCE (%)	AVT (%)	Bandgap (eV)	Ref
1.5% Cs (0.8 M) (average value)	0.91	0.83	0.61	0.46	61.62	2.92	This work
MAPbCl_3_ (champion value)	1.11	0.61	0.358	0.24	72.1	3.04	^29^
MAPbCl_2.4_Br_0.6_ (champion value)	1.13	0.85	0.435	0.42	73.0	2.83	^29^
MAPbCl_3_ (champion value)	1.01	0.57	0.3647	0.21	74.60	3.04	^34^

**Figure 7:**
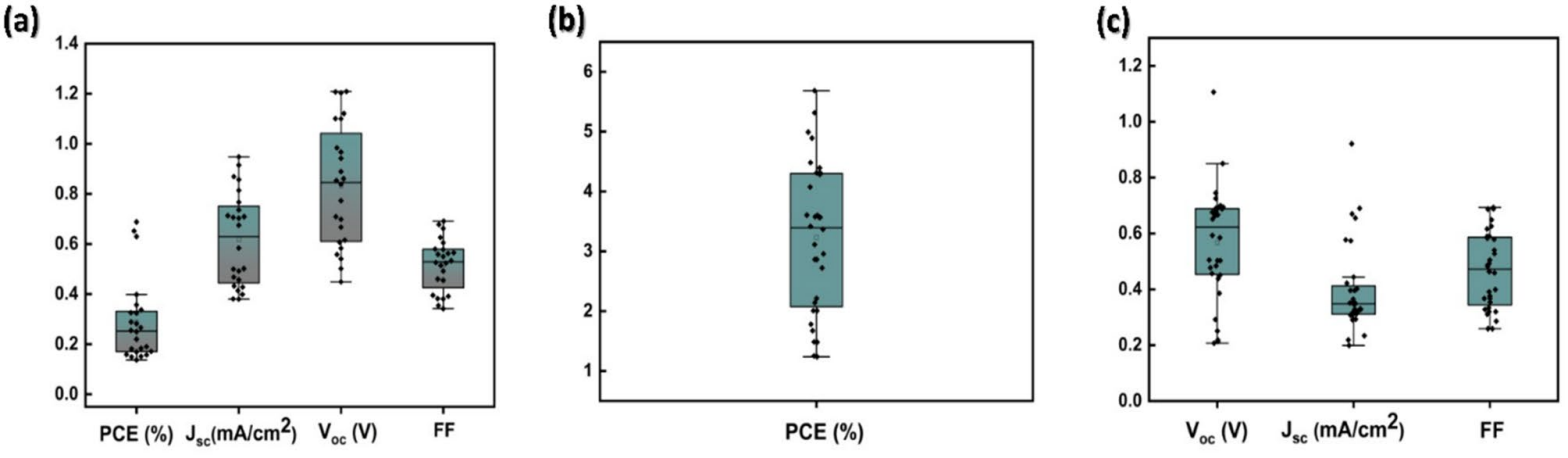
(**a**) Statistic box charts of 0.8 M, 1.5% Cs condition including PCE (%), J_sc_ (mA/cm^2^), V_oc_ (V), and FF tested under xenon lamp irradiation, (**b**) PCE statistic box chart of 0.8 M, 1.5% Cs condition tested under UV (365 nm), and (**c**) Statistic box charts of 0.8 M, 1.5% Cs condition including J_sc_ (mA/cm^2^), V_oc_ (V), and FF tested under UV (365 nm) irradiation.

Not only our fabricated transparent films have good efficiency under one sun irradiation (100 mW/cm^2^), but also have excellent performance under the irradiation of UV light (365 nm, 2.4 mW/cm^2^). Our best condition (0.8 M) has best PCE of 5.24% with V_oc_ of 1.14 V under UV light. This significant improvement in performance occurs due to the materials suitability of absorbing UV. Comparison between champion PV performance of 0.8 M, 1.5% Cs under the two different light sources is given in Table 3. The statistics box charts of the transparent perovskite solar cell performances under one sun and UV light (365 nm) are shown in Fig. 7a–c. Compilation of UV absorbance of a perovskite film (0.8 M, 1.5% Cs), UV-365 nm emission spectrum, and xenon lamp emission spectrum is shown in Fig. S10 of the supporting information. Comparison of UV power intensity between different types of lamps and real sun is given in Fig. S11, suggesting feasibility for practical usage. Raw data (Cs 1.5%, 0.8 M) of power conversion efficiency (PCE), short-circuit photocurrent density (J_sc_), open-circuit voltage (V_oc_), and fill factor (FF) tested under one sun irradiation (100 mW/cm^2^) and UV (365 nm, 2.4 mW/cm^2^) are given in Table S3 and Table S4. For clear perovskite thin films even with a suitable solvent, the surface is typically far from bead-like smooth morphology typically seen in dark perovskite films. Furthermore, the clear perovskite films are only 300 nm thick unlike the typical thicknesses between 500 and 1000 nm for dark perovskite films. As morphological variation is coupled with low thickness, more optoelectronic properties’ fluctuations are expected as seen in Fig. 7. The devices were kept inside the glovebox for 90 days, then measured for the stability performance as shown in Table S5. The instability is likely from the known problem of using Ag/Au electrode along with Spiro-OMeTAD. Device performances of different Cs contents with unoptimized device conditions are shown in Table S6.

**Table 3:** Champion PV performances of 0.8 M, 1.5% Cs under UV-365 nm and xenon lamp irradiation.

Light source	V_oc_ (V)	J_sc_ (mA/cm^2^)	FF	PCE (%)	AVT (%)	R_s_ (ohm.cm^2^)	R_sh_ (ohm.cm^2^)
Xenon lamp	1.10	0.99	0.63	0.69	61.62	232	143,417
UV (365 nm)	1.14	0.42	0.38	5.24	61.62	1081	9182

**Figure 8:**
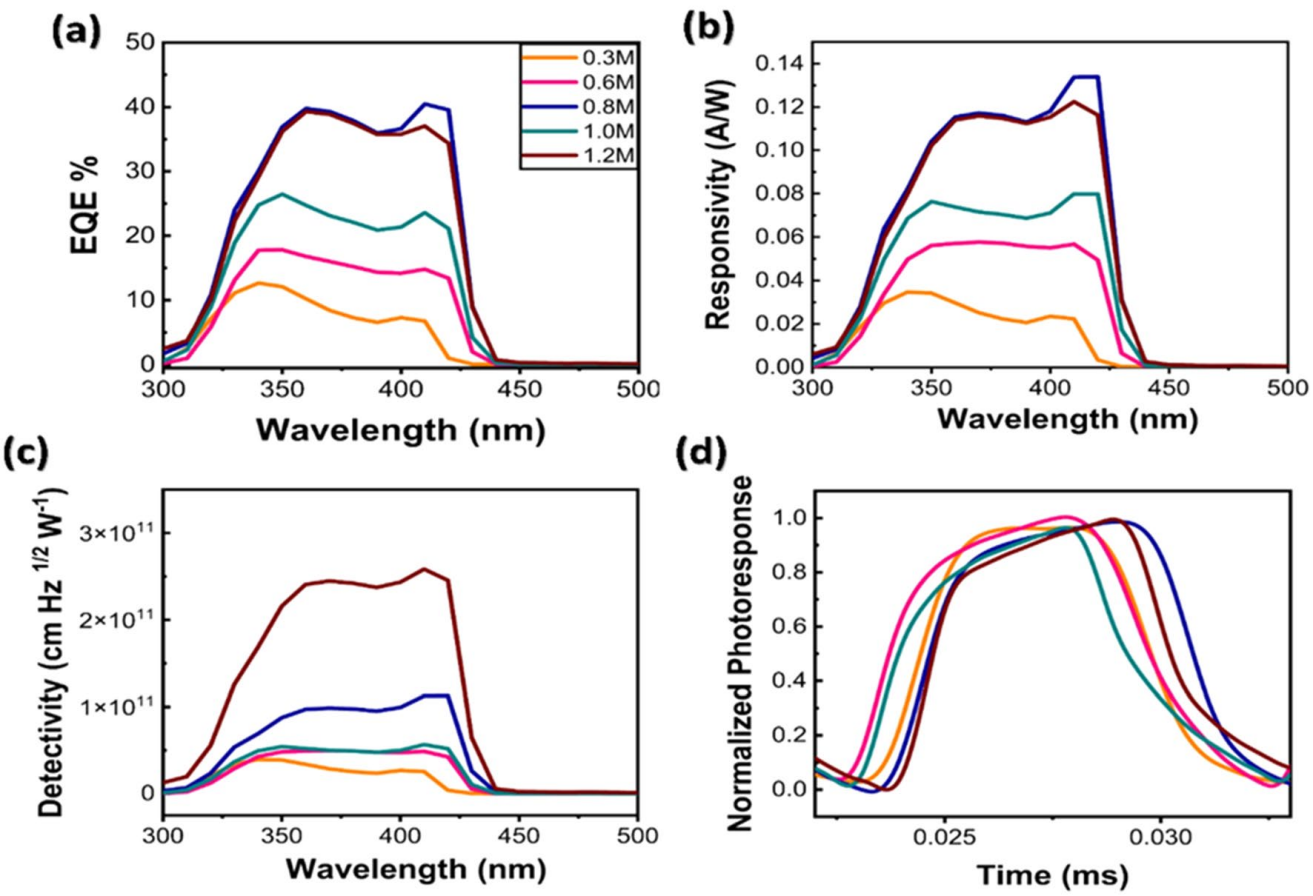
(**a**) EQE, (**b**) Responsivity, (**c**) Detectivity spectra, and (**d**) Normalized photo-responses of 1.5% Cs under 100 Hz optical chopper.

As stated above, the films with lower AVT and higher thicknesses are suitable for photodetector applications. The external quantum efficiency (EQE), responsivity (R), and specific detectivity (D*) within 300–900 nm, and characteristics of UV light detections are studied. EQE measures the ratio of electrons generated by a known number of photons^31,68^. Therefore, EQE reflects the relationship between light intensity and generated current. The detail explanations of EQE, R, and D* are carefully stated in our previous report^71^. In Fig. 8a, the highest EQE values were 40.47%, 37.05%, and 6.77% in 1.2 M, 0.8 M, and 0.3 M, respectively, having the same trend as the responsivity curves in Fig. 8b. At 365 nm, the EQE values were 39.54%, 39.02%, and 9.48% for 1.2 M, 0.8 M, and 0.3 M, respectively. Our EQE data strongly indicates that in the range of 300 nm to 430 nm, the films can detect higher amount of the photons compared to other detection ranges. As expected, the clear perovskite materials do not absorb any light after 450 nm. EQE data supports the bandgaps from UV–Vis data, the good solar cell performances under UV exposure, and aptness of 1.2 M, 1.5% Cs perovskite films for UV detector application.

For photodetectors, the specific detectivity is determined by main noise and light source in dark^72^. Figure 8c shows that the detectivity becomes higher when the AVT drops. The highest D* values were 2.58 × 10^12^, 1.12 × 10^12^, and 2.55 × 10^10^ (cm Hz^1/2^ W^-1^) for 1.2 M, 0.8 M, and 0.3 M, respectively. At 365 nm, the D* values were 2.42 × 10^11^, 9.48 × 10^10^, 3.12 × 10^10^ (cm Hz^1/2^ W^-1^) for 1.2 M, 0.8 M, and 0.3 M, respectively. The normalized photo-responses under 365 nm UV light of clear perovskite photodetectors under 100 Hz optical chopper are showed in Fig. 8d. As shown in Fig. 8d, 1.17 ms, 1.46 ms, and 2.41 ms were achieved from 0.3 M, 0.8 M, and 1.2 M for the rise time, while the fall time durations were 2.38 ms, 1.23 ms, and 2.50 ms, respectively. All of our samples demonstrate excellent photodetector performances in terms of τ_rise_ and τ_fall_ among other reported transparent photodetectors in Table S7. Our champion device can achieve the rise time of 2.41 ms and the fall time of 2.50 ms. The data including the EQE, R, D*, τ_rise_ and τ_fall_ are summarized in Table 4. The comparison graphs showing τ_rise_ and τ_fall_ of 1.5% Cs with concentration variation (0.3 M, 0.6 M, 0.8 M, 1.0 M, and 1.2 M) are shown in Fig. S12. The photo-responses with more cycles are given in Fig. S13.

**Table 4:** Comparison of photodetector parameters for different film thicknesses at 365 nm.

Concentration	τ_rise_ (ms)	τ_fall_ (ms)	On/off ratio	EQE (%)	Detectivity (cm Hz^1/2^ W^-1^)	Responsivity (A/W)
0.3 M	1.17	2.38	0.49	9.48	3.12 × 10^10^	0.027
0.6 M	2.09	2.27	0.92	16.58	4.94 × 10^10^	0.057
0.8 M	1.46	1.23	1.18	39.02	9.78 × 10^10^	0.116
1.0 M	1.73	1.90	0.91	23.86	5.10 × 10^10^	0.073
1.2 M	2.41	2.50	0.96	39.54	2.42 × 10^11^	0.115

## Conclusions

In summary, a clear, transparent, quasi-2D (*n* = 40) structure with the mixture of Cs/MA/FA was developed. The absorption spectral range was successfully tuned to 410–430 nm, unlocking potential usages for UV-current conversion and UV detection. The impacts of Cs incorporation into band alignment, morphology, and device performance are carefully studied. Low Cs content along with proper solvent choice results in higher absorbance and uniform morphology. The clear transparent perovskite solar cells achieved highest PCE of 0.69% under xenon lamp irradiation and 5.24% under UV light. Thicker transparent films show potential as a UV detector, exhibiting the EQE of 39.54%, detectivity of 2.42 × 10^11^ cm Hz^1/2^ W^−1^, and fast response of 2.41 ms in rise time and 2.50 ms in fall time at 365 nm. This work demonstrates the versatility of tuning perovskite compositions for desired optoelectronic applications.

## Supplementary Information

Supplementary Information.

## Data Availability

The datasets used and/or analysed during the current study available from the corresponding author on reasonable request.
