# Supplementary material for: Improving morphology and optoelectronic properties of ultra-wide bandgap perovskite via Cs tuning for clear solar cell and UV detection applications
Source: Sci Rep. 2023 Feb 20;13:2965. doi: 10.1038/s41598-023-29409-y (PMC9941583; doi:10.1038/s41598-023-29409-y)
Supplement: Supplementary file 1 — Supplementary Information. [file 41598_2023_29409_MOESM1_ESM.docx]

**Improving Morphology and Optoelectronic Properties of Ultra-wide Bandgap Perovskite via Cs Tuning for Clear Solar Cell and UV Detection Applications**

Myo Zin Tun^1^, Pimsuda Pansa-Ngat^1^, Pipat Ruankham^2,3^, Ko Ko Shin Thant^1^, Sirawit Kamnoedmanee^1^, Chaowaphat Seriwattanachai^1^, Worawut Rueangsawang^1^, Ratchadaporn Supruangnet^4^, Hideki Nakajima^4^, Pongsakorn Kanjanaboos^1,5^*

*^1^School of Materials Science and Innovation, Faculty of Science, Mahidol University, Nakhon Pathom 73170, Thailand.*

*^2^Department of Physics and Materials Science, Faculty of Science, Chiang Mai University, Chiang Mai 50200, Thailand.*

*^3^Research Center in Physics and Astronomy, Faculty of Science, Chiang Mai University, Chiang Mai 50200, Thailand.*

*^4^Synchrotron Light Research Institute (Public Organization), Nakhon Ratchasima 30000, Thailand.*

*^5^Center of Excellence for Innovation in Chemistry (PERCH‑CIC), Ministry of Higher Education, Science, Research and Innovation, Bangkok 10400, Thailand.*

**Pongsakorn.kan@mahidol.edu*

**Supplementary Information**

**
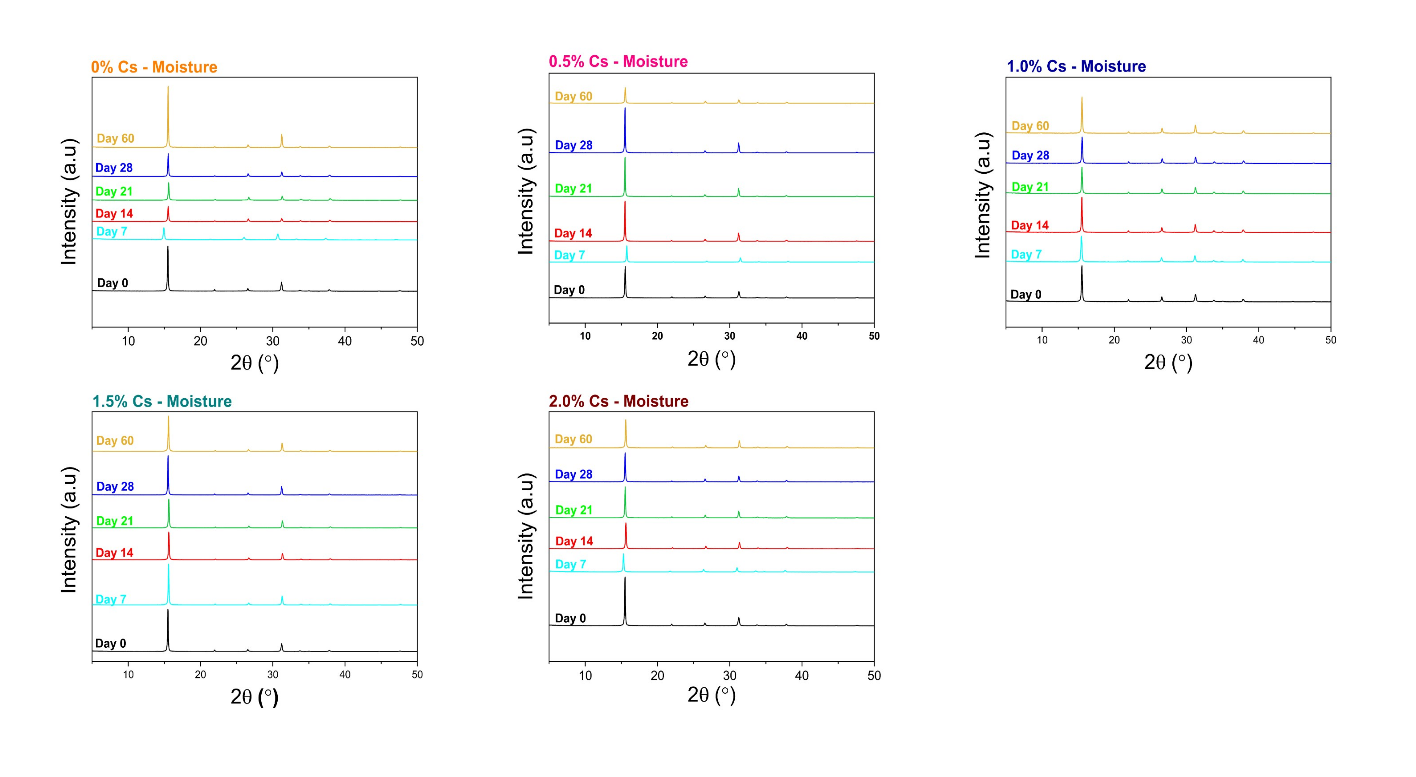
**

**Figure S1** Moisture XRD stability of (PEA)_2_(Cs_x_MA_0.61-x_FA_0.39_)_39_(Pb)_40_(Cl_0.88-0.32x_Br_0.12+0.32x_)_121_ with different Cs contents from 0% - 2.0% Cs (0.8 M).

**
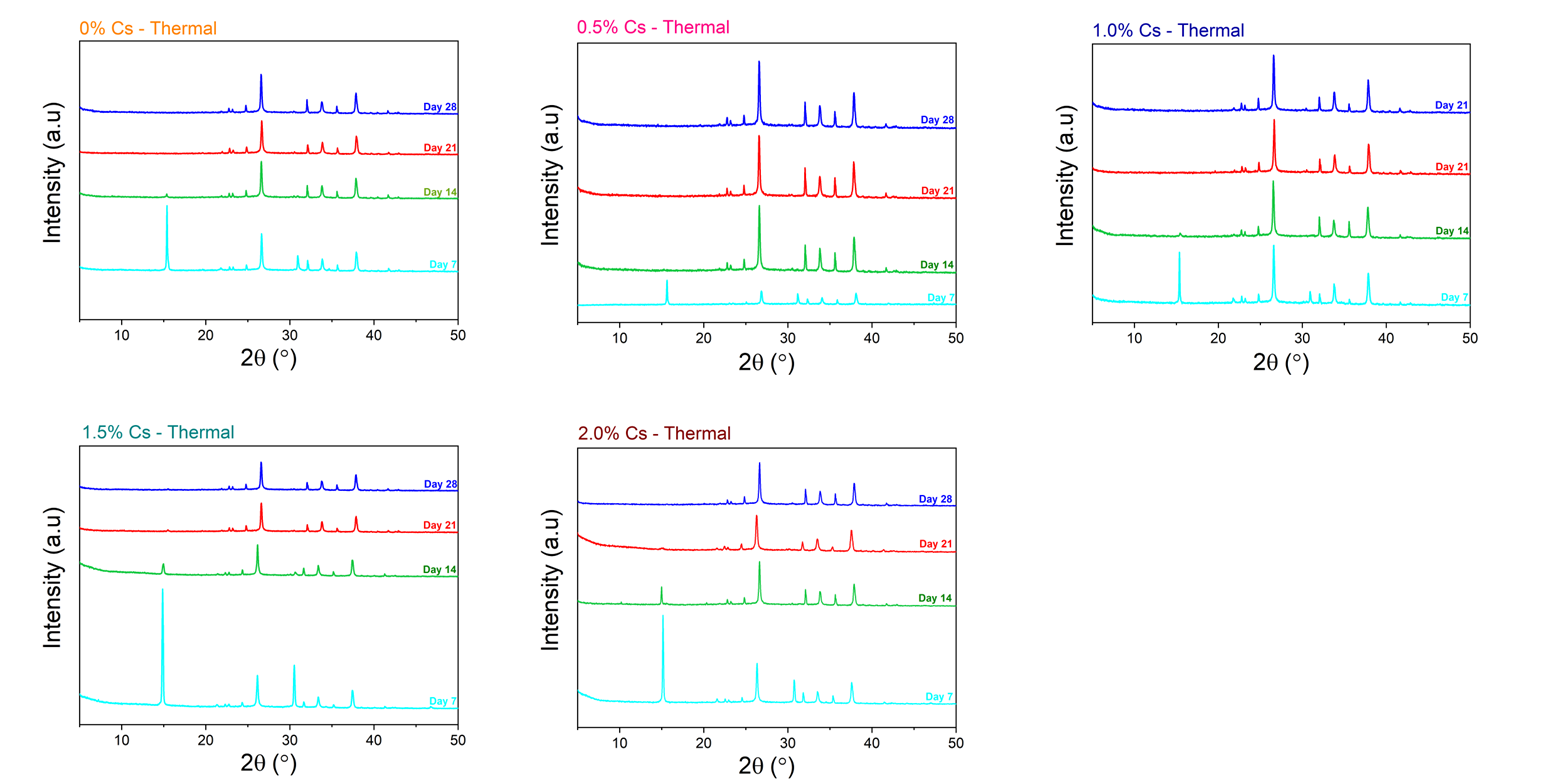
**

**Figure S2** Thermal XRD stability of (PEA)_2_(Cs_x_MA_0.61-x_FA_0.39_)_39_(Pb)_40_(Cl_0.88-0.32x_Br_0.12+0.32x_)_121_ with different Cs contents from 0% - 2.0% Cs (0.8 M).

**Table S1** UPS data of (PEA)_2_(Cs_x_MA_0.61-x_FA_0.39_)_39_(Pb)_40_(Cl_0.88-0.32x_Br_0.12+0.32x_)_121_ with different Cs contents
(0.8 M).

| **Condition** | **Work function Φ** | **Conduction band  minimum**  **(eV)** | **Valance band maximum**  **(eV)** | **Bandgap**  **(eV)** |
| --- | --- | --- | --- | --- |
| **0% Cs** | 3.96 | 3.16 | 6.09 | 2.93 |
| **0.5% Cs** | 4.06 | 3.29 | 6.23 | 2.94 |
| **1.0% Cs** | 3.94 | 3.38 | 6.29 | 2.91 |
| **1.5% Cs** | 4.27 | 3.94 | 6.86 | 2.92 |
| **2.0% Cs** | 4.06 | 3.96 | 6.89 | 2.93 |

**
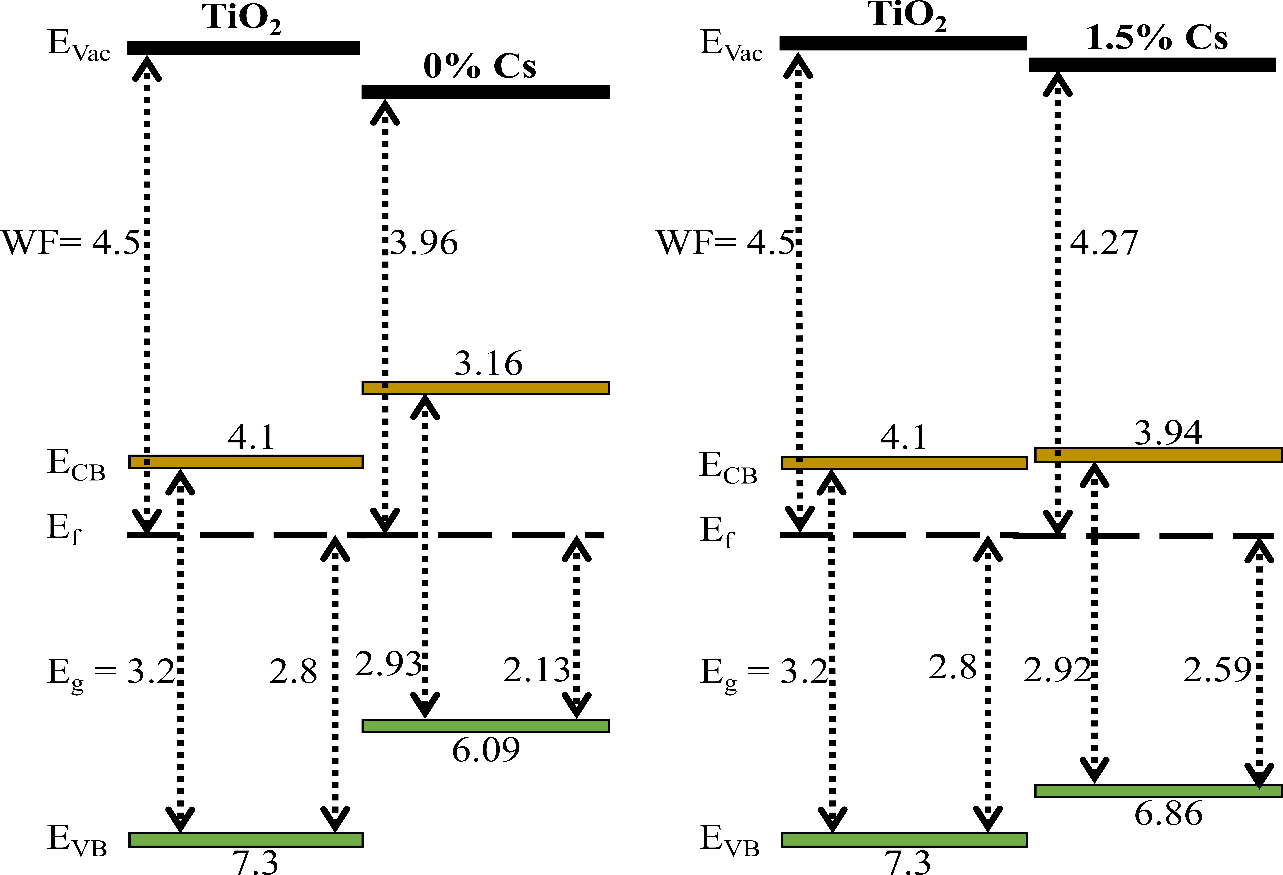
**

**Figure S3** Band alignment diagram of 1.5% Cs (0.8 M).

**
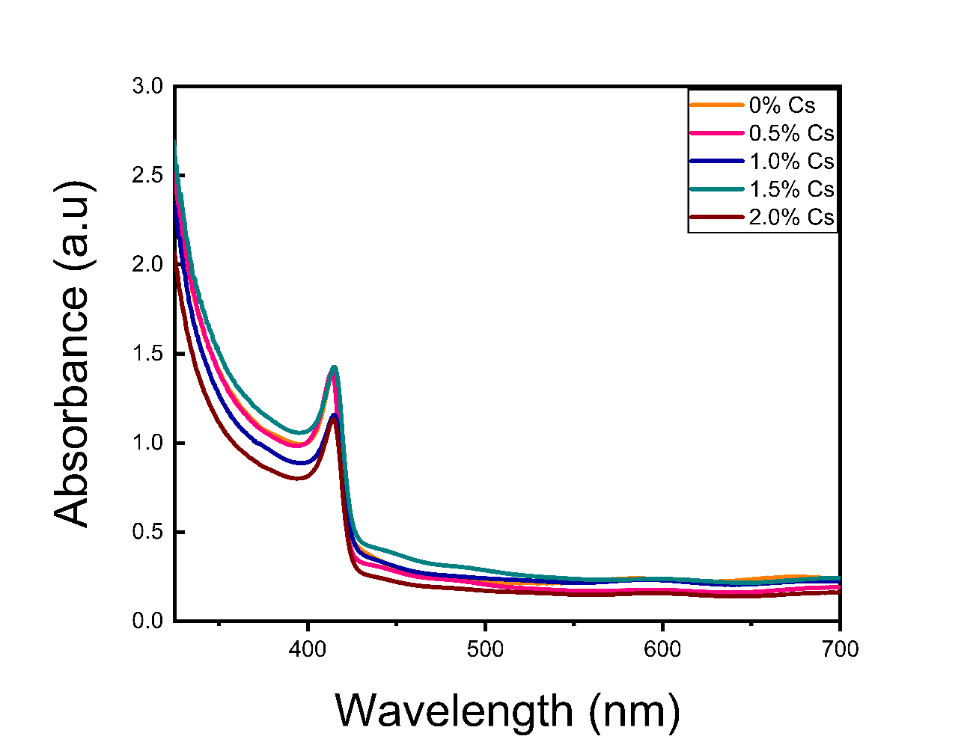
**

**Figure S4** UV absorbance spectra of (PEA)_2_(Cs_x_MA_0.61-x_FA_0.39_)_39_(Pb)_40_(Cl_0.88-0.32x_Br_0.12+0.32x_)_121_ from 0% - 2.0% Cs (0.8 M).

**
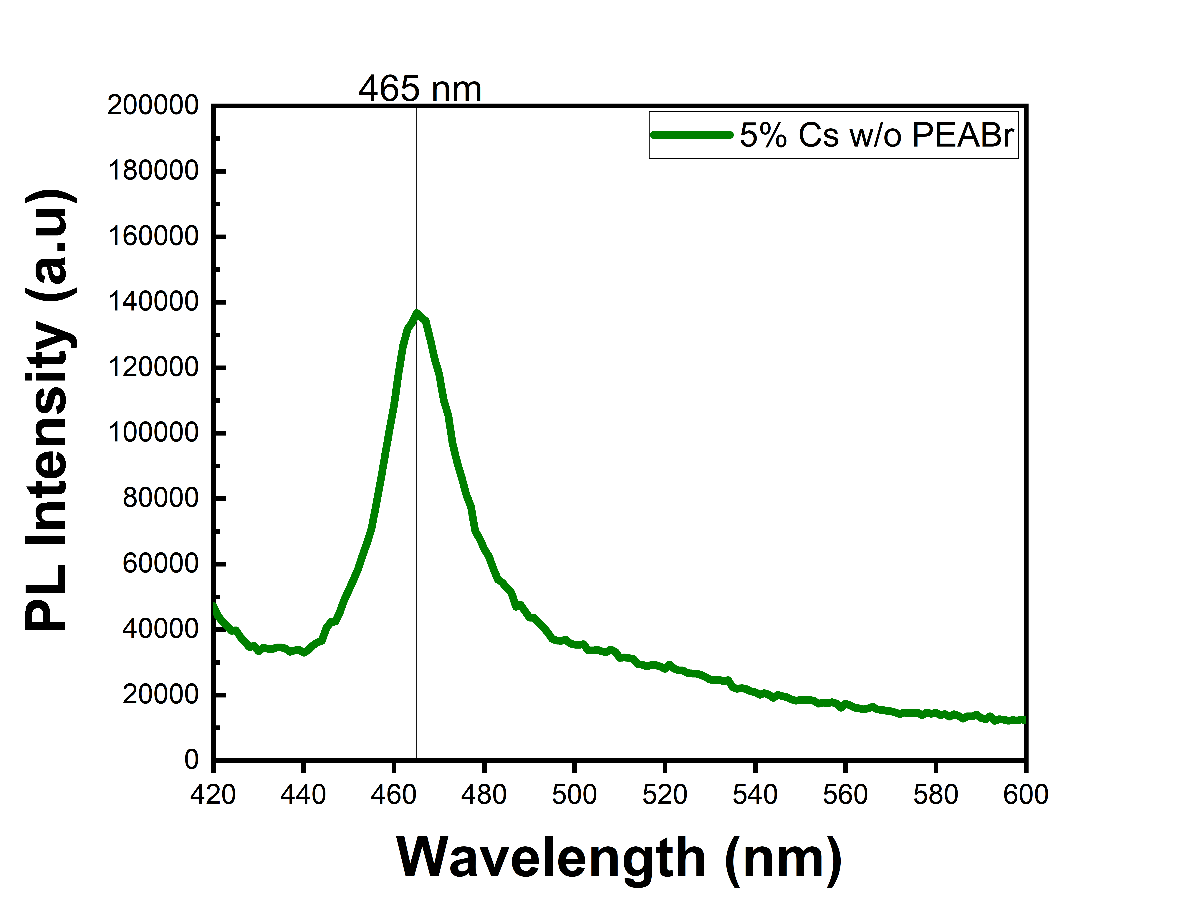
**

**Figure S5** PL spectra of 5% Cs perovskite film prepared in the condition without organic cation (PEABr) (0.5 M).

**
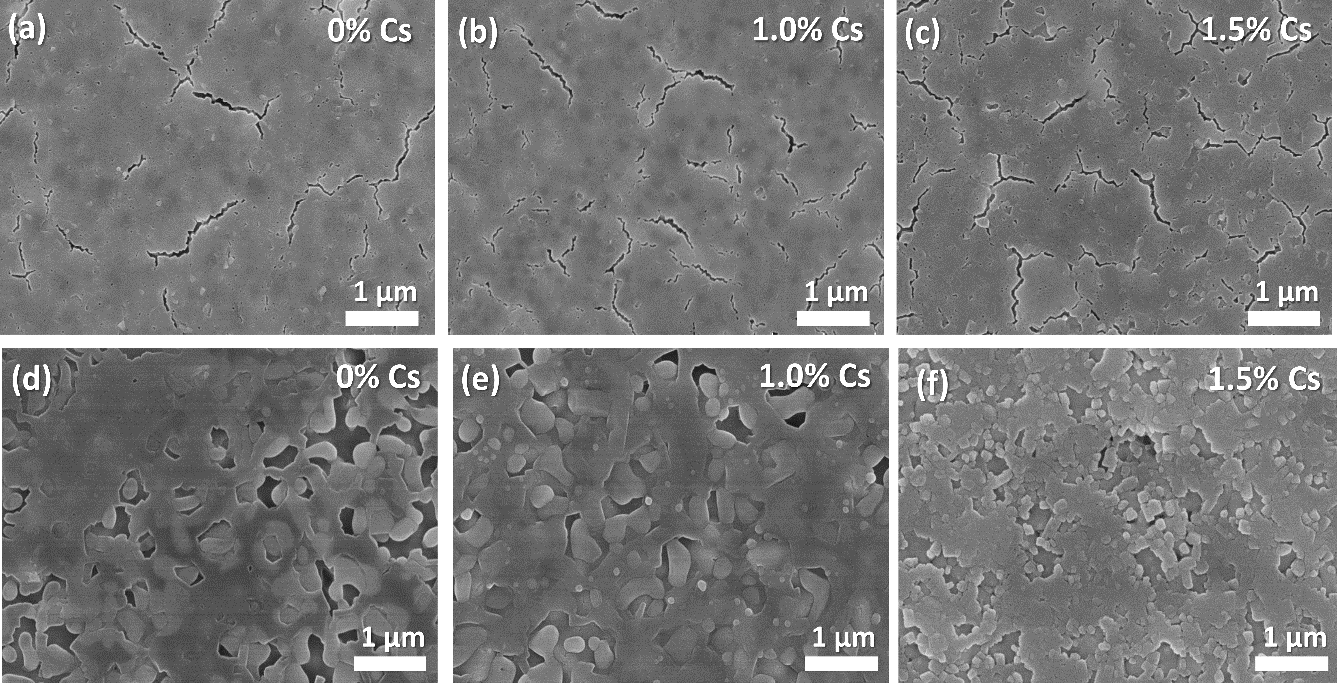
**

**
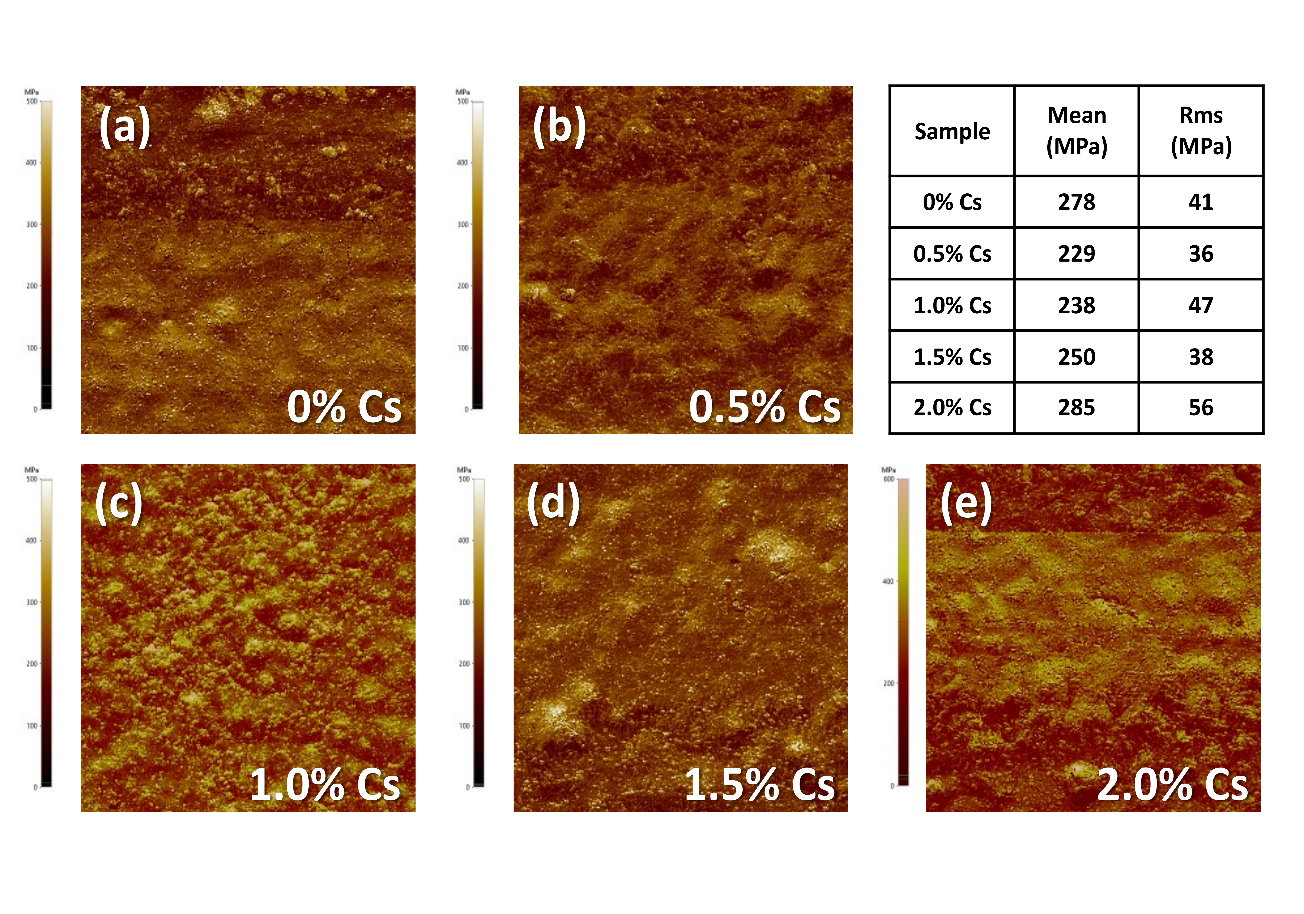
Figure S6** (a-c) SEM images of films prepared in DMF: DMSO (4:1) via different Cs contents (0% Cs, 1.0 % Cs, and 1.5% Cs, annealed at 100 °C), (d-e) SEM images of films prepared in DMSO via different Cs contents (0% Cs, 1.0 % Cs, and 1.5% Cs, annealed at 125 °C).

**Figure S7** AFM surface modulus mapping of (PEA)_2_(Cs_x_MA_0.61-x_FA_0.39_)_39_(Pb)_40_(Cl_0.88-0.32x_Br_0.12+0.32x_)_121_ from
0% - 2.0% Cs (0.8 M).

**Table S2** Solubility of different solvents.^1^

| **Type of Solvent** | **D_N_** | **Interaction with Cl^-^** |
| --- | --- | --- |
| **DMSO** | 29.8 | soluble |
| **DMF** | 26.6 | insoluble |
| **GBL** | 18.0 | insoluble |

**
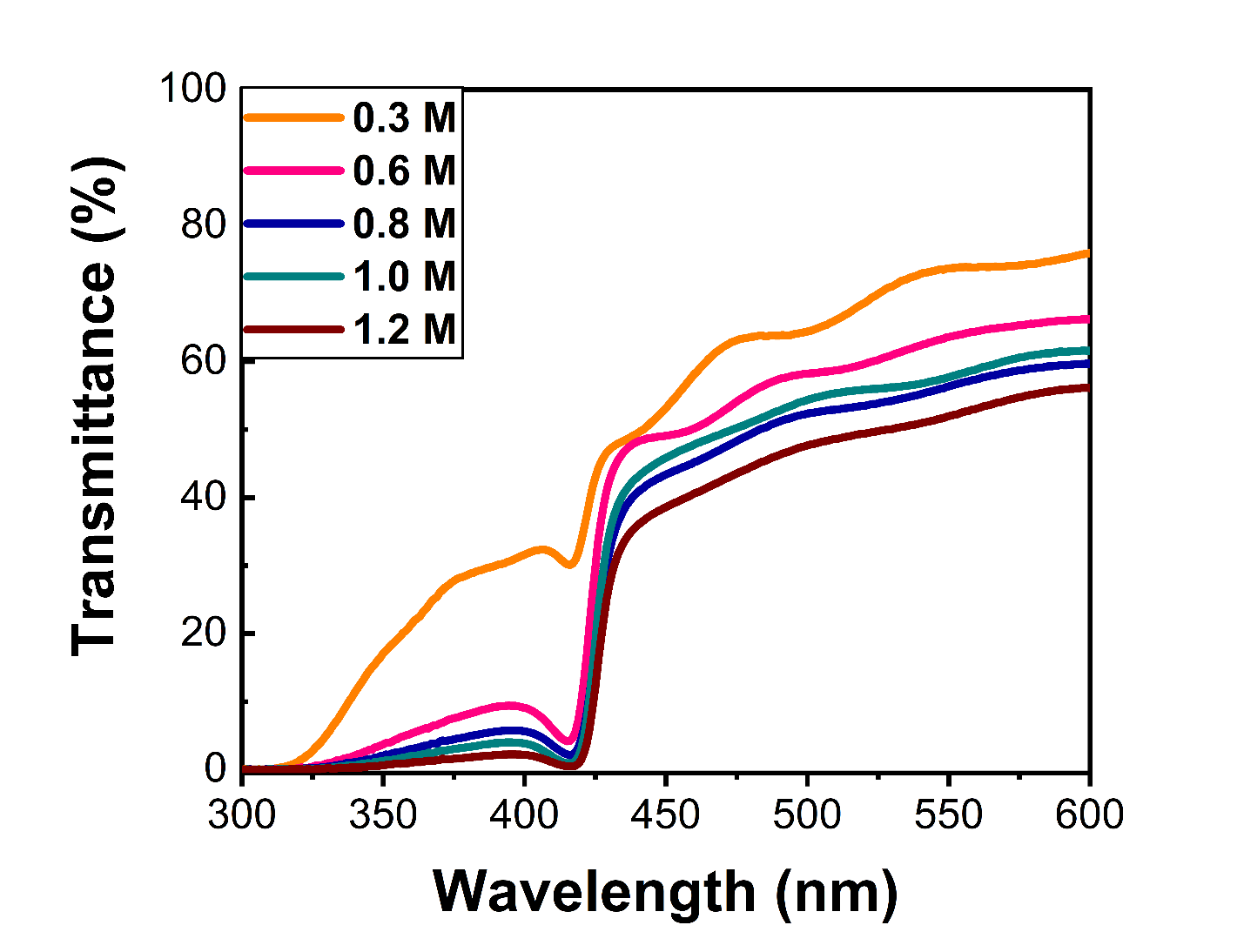
**

**Figure S8** UV transmittance spectra of 1.5% Cs with concentration variation (0.3 M, 0.6 M, 0.8 M, 1.0 M, and 1.2 M).

**
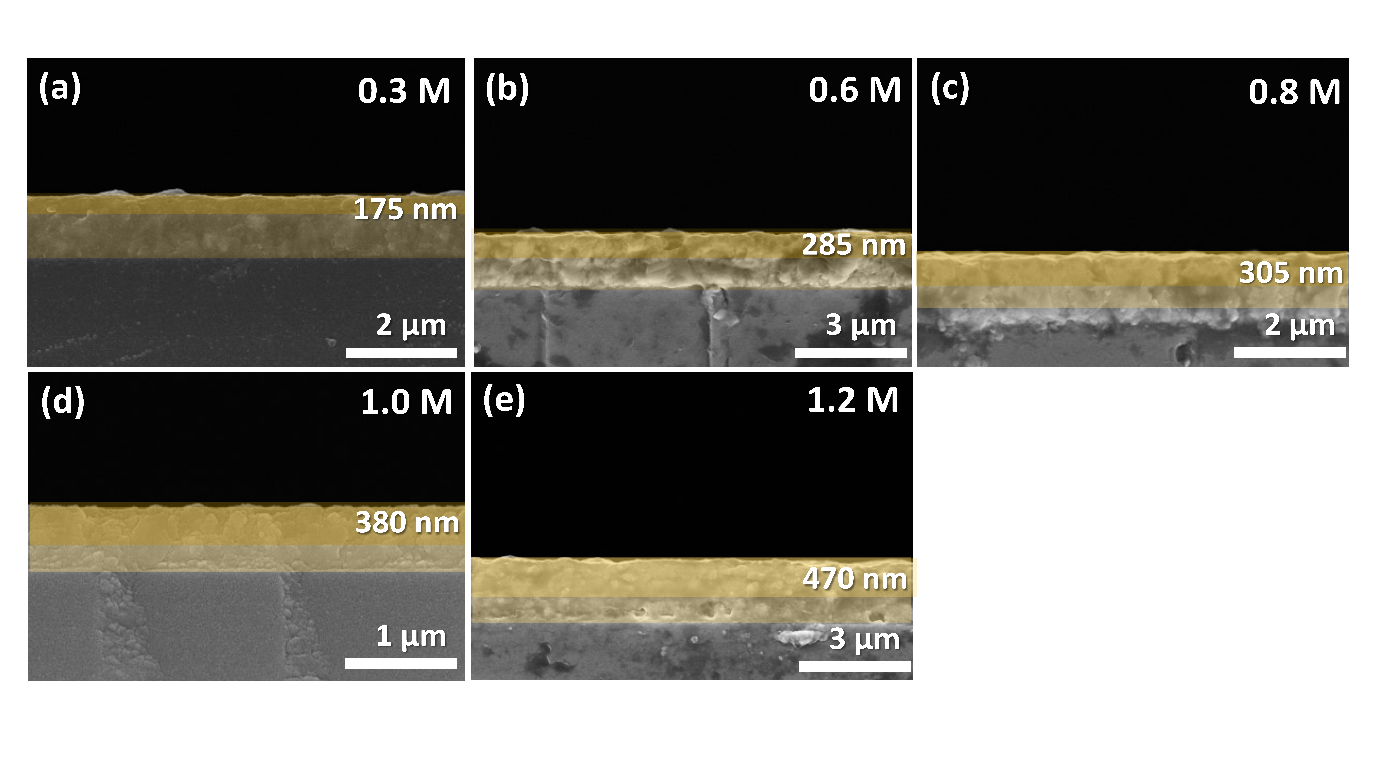
**

**Figure S9** FESEM cross-section images of 1.5% Cs with concentration variation (0.3 M, 0.6 M, 0.8 M, 1.0 M, and 1.2 M).

**Table S3** Raw data (Cs 1.5%, 0.8 M) of power conversion efficiency (PCE), short-circuit photocurrent density (J_sc_), open-circuit voltage (V_oc_), and fill factor (FF) tested under xenon lamp irradiation at 100 mW/cm^2^.

| **Sample** | **PCE (%)** | **J_sc_ (mA/cm^2^)** | **V_oc_ (V)** | **FF** |
| --- | --- | --- | --- | --- |
| Sample 1 | 0.357 | 0.857 | 0.984 | 0.391 |
| Sample 2 | 0.337 | 0.736 | 0.967 | 0.395 |
| Sample 3 | 0.688 | 0.948 | 1.100 | 0.578 |
| Sample 4 | 0.652 | 0.915 | 1.121 | 0.565 |
| Sample 5 | 0.630 | 0.869 | 1.101 | 0.580 |
| Sample 6 | 0.190 | 0.492 | 0.861 | 0.460 |
| Sample 7 | 0.183 | 0.468 | 0.773 | 0.514 |
| Sample 8 | 0.181 | 0.457 | 0.558 | 0.626 |
| Sample 9 | 0.159 | 0.428 | 0.699 | 0.533 |
| Sample 10 | 0.398 | 0.814 | 0.889 | 0.550 |
| Sample 11 | 0.149 | 0.675 | 0.449 | 0.492 |
| Sample 12 | 0.157 | 0.381 | 1.203 | 0.342 |
| Sample 13 | 0.326 | 0.706 | 1.209 | 0.381 |
| Sample 14 | 0.324 | 0.703 | 1.207 | 0.382 |
| Sample 15 | 0.250 | 0.713 | 0.667 | 0.526 |
| Sample 16 | 0.266 | 0.767 | 0.583 | 0.559 |
| **Sample** | **PCE (%)** | **J_sc_ (mA/cm^2^)** | **V_oc_ (V)** | **FF** |
| Sample 17 | 0.255 | 0.709 | 0.541 | 0.524 |
| Sample 18 | 0.289 | 0.584 | 0.607 | 0.455 |
| Sample 19 | 0.282 | 0.502 | 0.942 | 0.562 |
| Sample 20 | 0.137 | 0.432 | 0.839 | 0.354 |

**Table S4** Raw data (Cs 1.5%, 0.8 M) of power conversion efficiency (PCE), short-circuit photocurrent density (J_sc_), open-circuit voltage (V_oc_), and fill factor (FF) tested under UV irradiation (365 nm, 2.4 mW/cm^2^).

| **Sample** | **PCE (%)** | **J_sc_ (mA/cm^2^)** | **V_oc_ (V)** | **FF** |
| --- | --- | --- | --- | --- |
| Sample 1 | 3.416 | 0.402 | 0.593 | 0.459 |
| Sample 2 | 3.112 | 0.321 | 0.688 | 0.493 |
| Sample 3 | 4.892 | 0.291 | 1.106 | 0.486 |
| Sample 4 | 3.368 | 0.219 | 0.850 | 0.579 |
| Sample 5 | 4.991 | 0.574 | 0.477 | 0.583 |
| Sample 6 | 3.599 | 0.365 | 0.438 | 0.540 |
| Sample 7 | 3.577 | 0.351 | 0.484 | 0.505 |
| Sample 8 | 2.864 | 0.396 | 0.690 | 0.367 |
| Sample 9 | 4.313 | 0.349 | 0.503 | 0.59 |
| Sample 10 | 4.285 | 0.351 | 0.451 | 0.649 |
| Sample 11 | 4.482 | 0.330 | 0.676 | 0.482 |
| **Sample** | **PCE (%)** | **J_sc_ (mA/cm^2^)** | **V_oc_ (V)** | **FF** |
| Sample 12 | 3.604 | 0.325 | 0.505 | 0.528 |
| Sample 13 | 5.684 | 0.670 | 0.745 | 0.399 |
| Sample 14 | 4.313 | 0.349 | 0.503 | 0.590 |
| Sample 15 | 5.315 | 0.655 | 0.725 | 0.391 |
| Sample 16 | 2.140 | 0.312 | 0.680 | 0.354 |
| Sample 17 | 3.567 | 0.690 | 0.700 | 0.259 |
| Sample 18 | 4.394 | 0.921 | 0.585 | 0.286 |
| Sample 19 | 2.006 | 0.329 | 0.667 | 0.320 |
| Sample 20 | 2.721 | 0.422 | 0.688 | 0.328 |
| Sample 21 (one sun testing was not performed) | 5.236 | 0.420 | 1.142 | 0.380 |

**Table S5** Stability performance measurement samples (Cs 1.5%, 0.8 M) which were kept in N_2_ glovebox: power conversion efficiency (PCE), short-circuit photocurrent density (J_sc_), open-circuit voltage (V_oc_), and fill factor (FF) tested under xenon lamp irradiation at 100 mW/cm^2^. The instability is likely from the known problem of using Ag/Au electrode along with Spiro-OMeTAD.

| **Testing time** | **PCE (%)** | **J_sc_ (mA/cm^2^)** | **V_oc_ (V)** | **FF** |
| --- | --- | --- | --- | --- |
| Fresh | 0.364 | 0.72 | 0.98 | 0.41 |
| After 90 days | 0.108 | 0.47 | 0.80 | 0.38 |

**
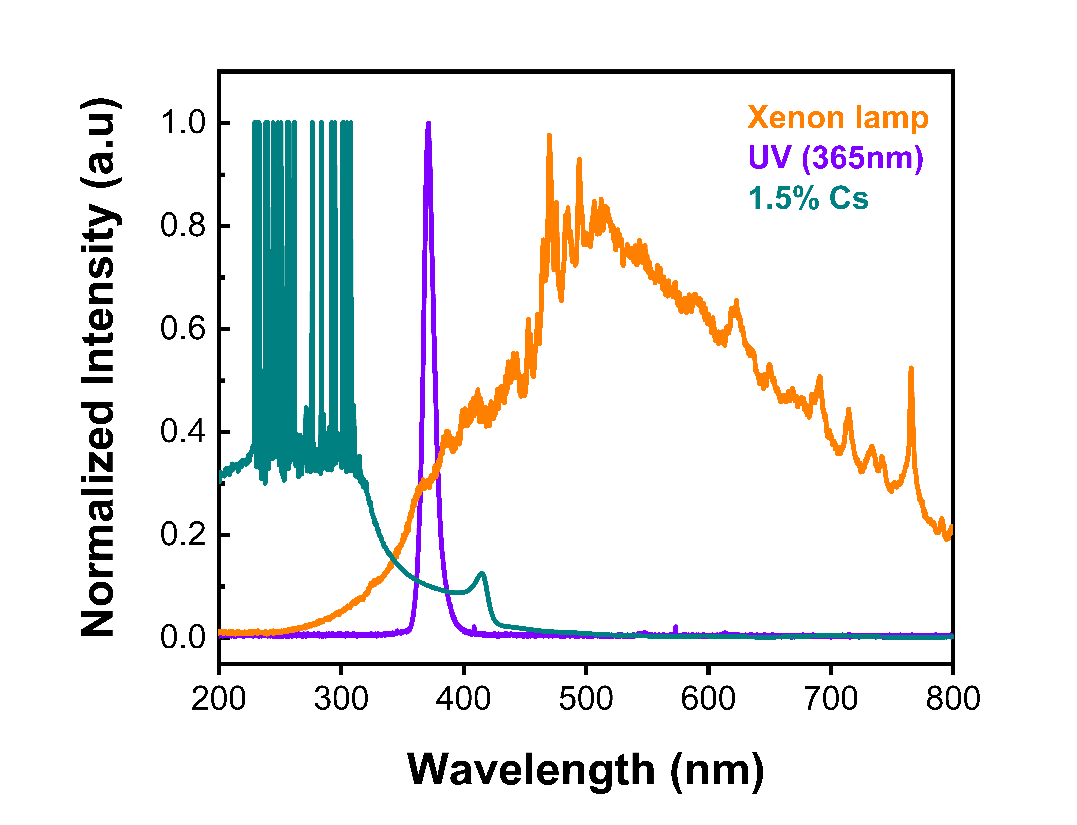
**

**Figure S10** Compilation of UV absorption spectrum of a perovskite thin film (0.8 M, 1.5% Cs), LLS-365 UV lamp spectrum (2.4 mW/cm^2^), and xenon lamp spectrum**.**

**
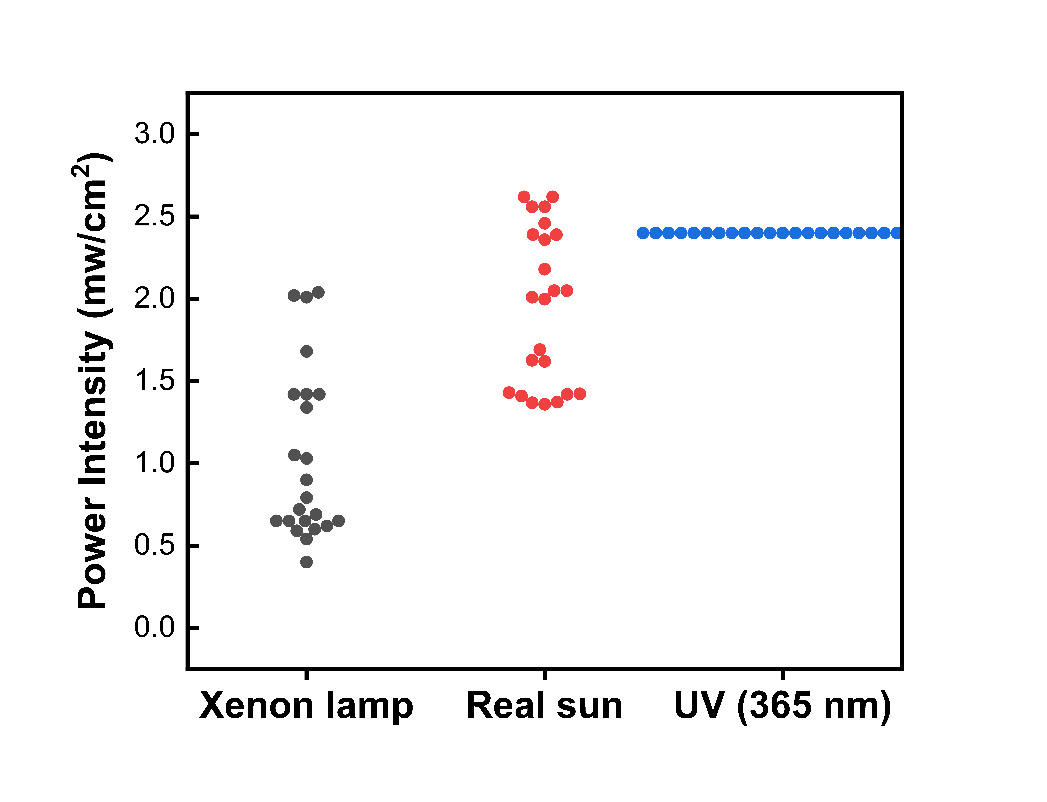
**

**Figure S11** Comparison of UV power intensities for different light sources.

**Table S6** Device performances of different Cs contents (0.8 M) tested under xenon lamp irradiation at 100 mW/cm^2^ (unoptimized device conditions).

| **Condition** | **PCE (%)** | **J_sc_ (mA/cm^2^)** | **V_oc_ (V)** | **FF** |
| --- | --- | --- | --- | --- |
| 0% Cs | 0.085 | 0.30 | 0.54 | 0.53 |
| 0.5% Cs | 0.055 | 0.21 | 0.43 | 0.39 |
| 1.0% Cs | 0.083 | 0.26 | 0.61 | 0.53 |
| 1.5% Cs | 0.180 | 0.56 | 0.59 | 0.42 |
| 2.0% Cs | 0.078 | 0.25 | 0.64 | 0.48 |

**Table S7** Comparison of different photodetector performances from clear perovskite absorbers.

| **Material** | **Method** | **Wavelength of detection**  **(nm)** | **R (A/W)** | **D* (cm Hz^1/2^ W^−1^)** | **EQE (%)** | **Ref** |
| --- | --- | --- | --- | --- | --- | --- |
| MAPbCl_3_ | Single crystal | 365 nm | 0.0469 | 1.2 x 10^10^ | - | 2 |
| MAPbCl_3_ | Solution | 398 nm | 0.071 | 1.2 x10^10^ | 23 | 3 |
| 1.5% Cs  (1.2 M) | Thin film | 365 nm | 0.115 | 2.42 x 10^11^ | 39.54 | This work |

**
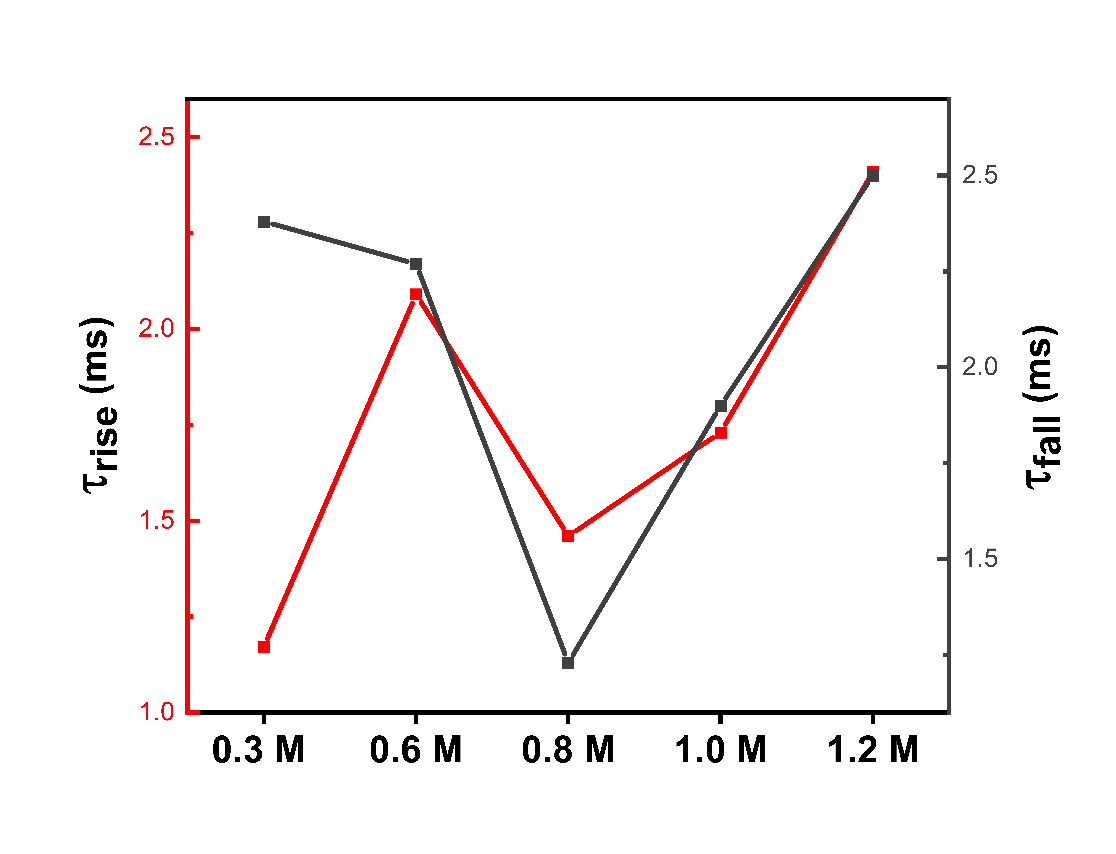
**

**Figure S12** τ_rise_ and τ_fall_ of 1.5% Cs with concentration variation (0.3 M, 0.6 M, 0.8 M, 1.0 M, and 1.2 M).


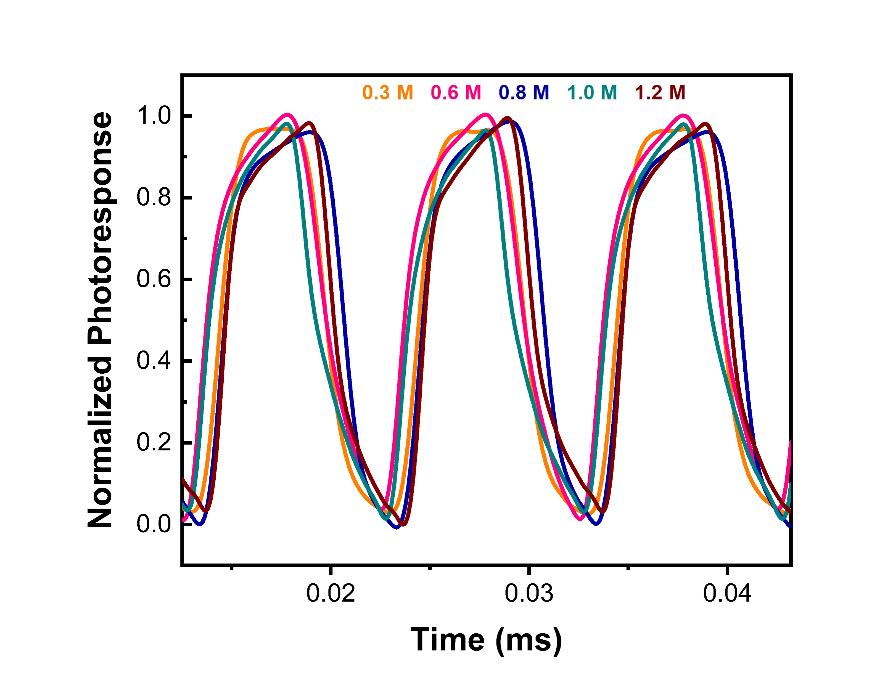


**Figure S13** Normalized photo-responses of 1.5% Cs under 100 Hz optical chopper and xenon lamp irradiation at 100 mW/cm^2^.

**References**

1. Petrov, A. A., Ordinartsev, A. A., Fateev, S. A., Goodilin, E. A. & Tarasov, A. B. Solubility of hybrid halide perovskites in dmf and dmso. *Molecules* **26**, 7541 (2021). doi: 10.3390/molecules26247541.

2. Zheng, E., Yuh, B., Tosado, G. A. & Yu, Q. Solution-processed visible-blind UV-A photodetectors based on CH_3_NH_3_Cl_3_ perovskite thin films. *Journal of Materials Chemistry C* **5**, 3796–3806 (2017). doi: 10.1039/C7TC00639J.

3. Shin, S. G., Bark, C. W. & Choi, H. W. Study on performance improvements in perovskite-based ultraviolet sensors prepared using toluene antisolvent and CH_3_NH_3_Cl. *Nanomaterials* **11**, 1000 (2021). doi: 10.3390/nano11041000.
